# Click Conjugates of Artificial Ribonucleases: Sequence Specific Cleavage with Multiple Turnover

**DOI:** 10.1002/chem.202500451

**Published:** 2025-05-06

**Authors:** Sandra Weber, Timo Weinrich, Ute Scheffer, Elisabeth Kalden, Michael W. Göbel

**Affiliations:** ^1^ Institute for Organic Chemistry and Chemical Biology Goethe University Frankfurt Max‐von‐Laue‐Strasse 7 D‐60438 Frankfurt am Main Germany

**Keywords:** aminobenzimidazole, heterocyclic guanidine, metal‐free catalyst, RNA cleavage, synthetic ribonuclease

## Abstract

The azido modified RNA cleaving catalyst **2** has been attached to oligonucleotides containing alkyne linkers in a central position. The resulting conjugates hybridize specifically with complementary RNA strands and cleave them with multiple substrate turnover. RNA half‐lives are in the range of 6–7 hours (pH 8, 37 °C). Some well‐placed locked nucleic acid (LNA) nucleotides can further increase substrate affinities and reaction rates significantly (*t*½ of 3.5 hours, *k*_obs_ = 0.20 h^−1^). RNA cleavage does not require metal ions and runs equally well in the presence of EDTA. Fragments of precisely defined lengths are formed, well suited for subsequent analysis by mass spectrometry and related bioanalytical techniques.

## Graphical abstract

A trisbenzimidazole catalyst was attached to central positions of DNA and DNA‐LNA oligonucleotides. The resulting conjugates bind complementary RNA strands and cleave them with multiple turnover and substrate half‐lives down to 3.5 hours. Up to 98.8% of cleavage occurs in a single site. The reaction does not depend on metal ions and also runs in the presence of EDTA.

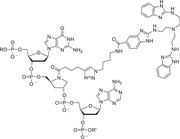

## 1 Introduction

Methods to cleave RNA site‐specifically ^[^ ^1^ ^]^ are equally important for gene silencing as well as for bioanalytical applications. Today, siRNAs ^[^ ^2^ ^]^ and gapmers ^[^ ^3^ ^]^ are the standard tools for the temporary knockdown of genes. Both approaches use cellular catalysts—Argonaute proteins and RNase H—which are programmed by synthetic oligonucleotides to eliminate specific mRNAs. More recently, Doudna has shown that Cas9 can serve the same purpose.^[^ ^4^ ^]^ RNA cleavage by ribozymes and deoxyribozymes is also well documented.^[^ ^5^ ^]^ All these systems have in common that natural biopolymers are the active catalysts. By complementing these approaches, chemists took up the challenge to develop fully synthetic RNA cleavers. Such compounds are mostly metal complexes containing Zn(II), Cu(II), or lanthanide ions.^[^ ^6^ ^]^ In form of oligonucleotide conjugates they can serve as artificial ribonucleases.^[^ ^7^ ^]^

**Figure 1:**
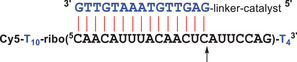
Typical oligonucleotide conjugate of trisbenzimidazole catalysts, bound to a complementary RNA strand. Cleavage occurs close to the duplex end (arrow). RNA nucleotides are shown in black, DNA in blue.

The mechanism of base induced RNA hydrolysis involves nucleophilic attack of the 2′ hydroxy group at phosphorus running through pentacoordinated transition states and forming finally a 2′,3′ cyclic phosphate. General bases to activate the nucleophile and general acids to protonate phosphoranes or leaving groups fundamentally contribute to catalysis. The example of RNase A shows that this kind of proton transfer can be perfectly mediated by the histidine and lysine residues of the enzyme. Thus, in addition to metal complexes, a number of metal‐free RNA cleavers has been developed in recent years,^[^ ^1^, ^8^ ^]^ for example tris(2‐aminobenzimidazoles), ^[^ ^9^ ^]^ and other guanidine analogs.^[^ ^10^ ^]^ Such catalysts can be attached to the 5′ end of DNA oligonucleotides (typically 15mers) or of DNA‐LNA mixmers (Figure 1).^[^ ^11^ ^]^ Phosphoramidite building blocks in combination with a manual coupling protocol have allowed us to prepare a larger number of conjugates in high yield.^[^ ^12^ ^]^ They hybridize with complementary RNA and hold catalyst and substrate in close proximity. With GC as the closing base pair, substrate half‐lives are in the range of 10–15 hours (pH 8.0, 37 °C) and up to 89% of cleavage occurs directly at the duplex end. Replacement of selected deoxynucleotides by LNA building blocks can accelerate the reaction by a factor of 3. In the 5′ terminal position, however, LNA has a detrimental effect, both in terms of rates and specificity. When the closing base pair is AT, cleavage slows down a bit and 4–5 fragments around the duplex end are formed. LNA building blocks again are helpful.

The major disadvantage of 5′ conjugates—when used under isothermal conditions—is a lack of multiple substrate turnover: The number of base pairs is not diminished in the cleavage step and the RNA fragments tend to stick to the conjugates. To overcome this type of product inhibition, the catalyst can be placed in the duplex center opposite to a bulge of the RNA strand. The resulting shorter cleavage fragments will quickly dissociate from the conjugate.^[^ ^13^ ^]^ In the present study, we have adopted this concept to trisbenzimidazole catalysts by using copper‐promoted click reactions. The resulting conjugates cleave their substrates with multiple turnover, high precision, and reaction rates at least comparable to those of related 5′ conjugates.

## 2 Results and Discussion

**Figure 2:**
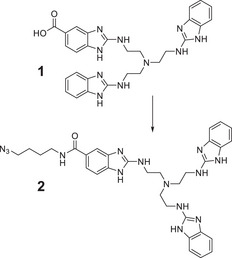
Attachment of an azide side chain to the trisbenzimidazole catalyst: 4‐azidobutylamine, **1**, DIC, DIPEA, DMF, HOBt, room temperature overnight (17%). DIC, N,N′‐Diisopropylcarbodiimide; DIPEA, N,N‐Diisopropylethylamine; DMF, Dimethylformamide; HOBt, 1‐Hydroxybenzotriazole.

**Figure 3:**
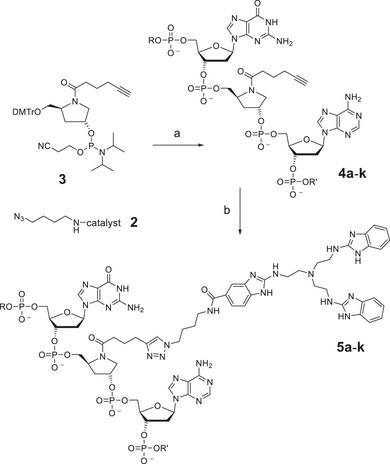
a) Oligonucleotides **4a**‐**k** were assembled on an Expedite 8909 synthesizer using standard coupling protocols (1 µmol scale), deprotected and purified by RP‐HPLC. b) CuSO_4_, TBTA, sodium ascorbate, 8.5 M urea in TEAA buffer pH 7.0, azide **2**, room temperature, 4 days under Ar. TBTA, tris(benzyltriazolylmethyl)amine; TEAA, triethylammonium acetate.

For the click reaction, an azide modified catalyst **2** was required and prepared from 4‐azidobutylamine and compound **1** ^[^ ^12b^ ^]^ (17%; Figure 2). To install an alkyne moiety in the oligonucleotide, we tested several linkers with C‐C triple bonds. Good results were obtained with the commercial phosphoramidite **3**. A series of oligonucleotide strands **4a**‐**k** was prepared from **3** by standard solid phase synthesis (Figure 3 and Table 1), purified and converted into the conjugates **5a**‐**k** by copper‐promoted dipolar cycloaddition. No protection of the benzimidazoles was required in this step. However, Cu(I) can be oxidized under air. This Fenton‐type reaction forms hydroxide radicals and may cause a degradation of DNA. The conjugation was therefore conducted under argon in the presence of TBTA stabilizing the Cu(I) oxidation state. All conjugates were again purified by RP‐HPLC. When an excess of TBTA was used, mass spectra of the purified conjugates indicated the absence of copper ions (Figures S38‐S56).

**Table 1:** Cleavage of RNA **6**. Substrate half‐lives and product distribution under saturation conditions (excess of conjugate).

Conjugate^[a]^	*t*_1/2_ [hours]^[b]^	*n*−1 [%]	*n* [%]	*n* + 1 [%]
^3′^ CGGCTGA X′ GGCTAG ^5′^ **5a**	7.1	7.5	91.6	0.1
^3′^ CGGCT GA X′ GGCTAG ^5′^ **5b**	7.7	1.5	97.9	0.0
^3′^ CGGCTG A X′ GGCTAG ^5′^ **5c**	12.3	1.5	98.5	0.0
^3′^ CGGCTGA X′ G GCTAG ^5′^ **5d**	15.1	25.4	74.6	0.0
^3′^ CGGCTGA X′ G G CTAG ^5′^ **5e**	6.7	11.5	88.5	0.0
^3′^ CGGCT G A X′ G G CTAG ^5′^ **5f**	6.4	3.5	96.0	0.0
^3′^ C G GCT G A X′ G G C T A G ^5′^ **5g**	7.0	3.2	96.2	0.0

^[a]^ DNA blue, LNA red. X′: hydroxyprolinol with catalyst attached. General conditions: 37 °C; 50 mM Tris buffer pH 8.0, 100 mM NaCl; 150 nM **6**, 10 equiv. of **5**.

^[b]^ Experimental error ± 10%.

**Figure 4:**
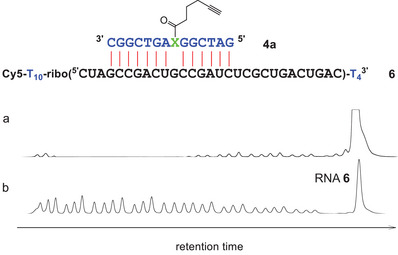
a) In‐line probing of RNA **6** (150 nM) in the presence of compound **4a** (1.5 µM; 50 mM Tris buffer (pH 8.3), 20 mM MgCl_2_, 100 mM KCl, 37 °C, 20 hours). Suppressed cleavage in positions 4–17 is caused by hybridization. b) The hydrolysis ladder of RNA **6** with 29 dye‐labeled fragment peaks. RNA nucleotides are shown in black, DNA in blue. X stands for hydroxyprolinol.

The dye‐labeled synthetic model RNAs **6**–**8** and the dye‐unlabeled 412mer transcript **9** ^[^ ^11b^ ^]^ were used as substrates in the cleavage experiments. The short model RNAs are embedded into a series of deoxynucleotides and labeled with a fluorescent cyanine dye. Benzimidazole catalysts cleave the RNA part selectively. When analyzed by gel electrophoresis in an ALFexpress sequencer, only the dye‐labeled fragments are visible. Figure 4 shows in‐line probing ^[^ ^14^ ^]^ of RNA **6** (150 nM) in the presence of DNA **4a** (1.5 µM). The high Mg^2+^ concentration (20 mM) causes partial cleavage in any ribonucleotide position free to adopt the in‐line orientation of 2′‐OH, phosphorus, and the 5′‐O of the neighbor nucleotide. In duplex regions, the corresponding angle is far from 180° and cleavage is prevented. Accordingly, the 29mer RNA **6** produced 29 fragment peaks in the electropherogram (Figure 4b). DNA **4a** was expected to hybridize with ribonucleotides 4–17 (**4a·6**: *T_m_* = 47 °C; see Figure S11) and to suppress cleavage in this helical region. The resulting footprint (peaks 4–17) is clearly visible in Figure 4a. In this duplex G(11) may either form a bulge or stay within the stack of nucleobases. The low cleavage yield between U(10) and G(11), however, indicates a predominance of the stacked conformation.

**Figure 5:**
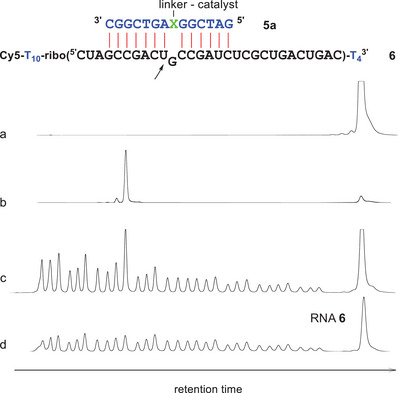
Conjugate **5a** cleaves model RNA **6** specifically in the single nucleotide bulge between U(10) and G(11). a) RNA **6** before incubation. b) RNA **6** (150 nM) after incubation with conjugate **5a** (1.5 µM) for 20 hours at 37 °C (50 mM Tris buffer pH 8, 100 mM NaCl). c) Hydrolysis ladder of RNA **6**, spiked with the cleavage fragments. d) Hydrolysis ladder of RNA **6**. RNA nucleotides are shown in black, DNA in blue. X stands for hydroxyprolinol.

The corresponding cleavage experiments with conjugate **5a** (0–2.25 µM) and RNA **6** (150 nM) were conducted at 37 °C in Mg^2+^‐free TRIS buffer (50 mM, pH 8.0). The electropherogram of the purified RNA showed a single peak before incubation (Figure 5, lane a). After 20‐hour reaction time, the product peaks (lane b) were identified by comparison with the hydrolysis ladder (lanes b‐d) and quantified by integration. Cleavage yields rose with increasing concentration of conjugate **5a** but reached saturation at 600 nM of **5a**, indicating quantitative hybridization of RNA **6** (Figure S12). Under saturation conditions (1.5 µM **5a**) the reaction followed first‐order kinetics with a substrate half‐life of 7.1 hours (Figure S22). 91.6% of cleavage occurred between U(10) and G(11) (indicated as “*n*” in Table 1), 7.5% between C(9) and U(10) (“*n*−1” in Table 1) and 0.1% between G(11) and C(12) (“*n* + 1”). Thus, compared to the 5′ conjugates of trisbenzimidazoles, RNA cleavage by conjugate **5a** is distinctly faster and slightly more precise. The question of multiple turnover is discussed below.

The influence of temperature was determined by incubating RNA **6** with conjugate **5a** for 4 hours at different conditions: 4 °C (5.4% cleavage), 25 °C (18.3%), 37 °C (31.4%), 50 °C (20.1%), and 60 °C (2.5%; Figure S36). Although the *T_m_* value for **5a·6** cannot be measured in the usual way, the corresponding value for **4a·6** suggests that it may be well below 50 °C. This explains the smaller extent of cleavage at increased temperatures.

To test the influence of LNA nucleotides, we synthesized a series of conjugates **5b**‐**g**, coinciding in sequence with **5a**, and conducted cleavage experiments with RNA **6** as shown above. Placing the LNA in the 3′ position next to X in conjugate **5c** slowed down the reaction. On the other hand, more than 98% of cleavage occurred in a single position between U(10) and G(11) (Table 1). Adding a pair of LNAs 3′ next to X (conjugate **5b**) almost restored the reactivity of **5a** without losing much of the precision. Placing the LNA in the 5′ proximity induced more cleavage in the *n*−1 position (conjugates **5d** and **5e**) and also doubled the half‐live of RNA **6** (conjugate **5d**). As well as **5b**, conjugate **5f** showed a good compromise of fast and precise cleavage. Adding more LNA building blocks in conjugate **5g** had no beneficial effect.

**Figure 6:**
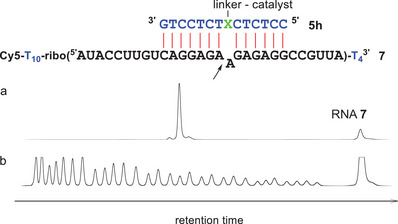
a) RNA **7** (150 nM) after incubation with conjugate **5h** (1.5 µM) for 20 hours at 37 °C (50 mM Tris buffer pH 8, 100 mM NaCl). b) Hydrolysis ladder of RNA **7**. RNA nucleotides are shown in black, DNA in blue. X: hydroxyprolinol.

A different site in RNA **7** was addressed by oligo **4h**. In‐line probing of this RNA (150 nM) in the presence of **4h** (1.5 µM) led to a similar footprint as shown in Figure 4: RNA cleavage was suppressed in all 14 positions involved in base pairing (Figure S8). The corresponding conjugate **5h** effectively cleaved RNA **7**, generating a fragment pattern comparable to that of RNA **6** and **5a** (Figure 6, Table 2). As before, the reaction of **7** and **5h** showed rate saturation with increasing conjugate concentrations and obeyed first order kinetics. With a substrate half‐life of 5.7 hours, **5h** reacted slightly faster compared to conjugate **5a**.

**Table 2:** Cleavage of RNAs **7** and **8**. Substrate half‐lives and product distribution under saturation conditions (excess of conjugate).

Conjugate^[a]^		*t*_1/2_ [hours]	*n*−1 [%]	*n* [%]	*n* + 1 [%]
^3′^ GTCCTCT X′ CTCTCC ^5′^	**5h**	5.7	3.3	90.7	6.0
^3′^ G T TG T A X′ A T GTT G AG ^5′^	**5j**	4.6	1.2	98.8	0.0
^3′^ G T T G TA X′ AT G T T GAG ^5′^	**5k**	3.5	1.2	96.8	1.0

^[a]^ DNA blue, LNA red, hydroxyprolinol with catalyst attached green. General conditions: 37 °C; 50 mM Tris buffer pH 8.0, 100 mM NaCl; 150 nM **7** or **8**, 1.5 µM **5**.

**Figure 7:**
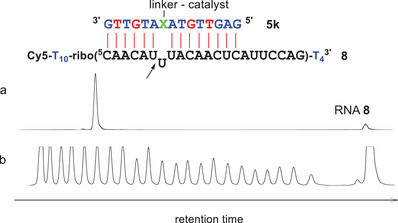
a) RNA **8** (150 nM) after incubation with conjugate **5k** (1.5 µM) for 20 hours at 37 °C (50 mM Tris buffer pH 8, 100 mM NaCl). b) Hydrolysis ladder of RNA **8**. RNA is shown in black, DNA in blue, LNA in red. X: hydroxyprolinol.

The sequence of RNA **8** was taken from the mRNA of the proto‐oncogenic kinase PIM1.^[^ ^11b^ ^]^ However, no footprint at all was visible in the in‐line probing of **8** (150 nM) in presence of oligonucleotide **4i** (1.5 µM, ^3′^ GTTGTA X ATGTTGAG ^5'^), indicating a lack of hybridization (Figure S10). To increase the duplex stabilities, two DNA‐LNA mixmers **4j** and **4k** were synthesized. In‐line probing with RNA **8** now showed the expected footprint (Figure S9). Interestingly, increased cleavage occurred in position 7 between U(7) and U(8) (compare with Figure 7). The stack of three consecutive uridines seems to be less stable and allows these nucleotides to form a bulge. The related conjugates **5j** and **5k** were highly active, cleaving their substrate between U(6) and U(7) with excellent specificity (Table 2). Both conjugates showed rate saturation (Figures S20‐S21) and followed first‐order kinetics (Figures S30‐S31). With a substrate half‐life of 3.5 hours, compound **5k** was also the fastest RNA cleaver in the present study. Addition of 1 mM of EDTA to the experiments did neither change the reaction kinetics of **5k** nor the cleavage pattern (Figure S32): The trisbenzimidazole acts as an organocatalyst void of metal ions. Hybridization of RNA and catalyst conjugates is a precondition for cleavage. Conjugate **5g**, for example is specific for RNA **6** and does not attack substrates **7** and **8** (Figures S33‐S35).

Eight out of ten conjugates have been incubated with an excess of their complementary RNAs (8.4–11.1 equivalents) for 95 hours. Multiple substrate turnover was observed in all cases (Table 3). The degree of cleavage correlated with the substrate half‐lifes reported in Tables 1 and 2. The most effective conjugate **5k** was able to cut almost 97% of RNA **8** when present in more than tenfold excess. Obviously, the presence of several LNA building blocks in compounds **5g**, **5j,** and **5k** did not affect the dissociation of cleavage fragments from the conjugates.

**Table 3:** Cleavage experiments with an excess of RNA show multiple substrate turnover

Conjugate^[a]^		*t*_1/2_ [hours]	Equiv. of RNA	Cleaved RNA [%]	Uncleaved RNA [%]
^3′^ CGGCTGA X′ GGCTAG ^5′^	**5a**	7.1	9.0	82.3	17.7
^3′^ CGGCT GA X′ GGCTAG ^5′^	**5b**	7.7	9.4	64.9	35.1
^3′^ CGGCTG A X′ GGCTAG ^5′^	**5c**	12.3	9.0	57.5	42.5
^3′^ CGGCT G A X′ G G CTAG ^5′^	**5f**	6.4	8.4	90.3	9.7
^3′^ C G GCT G A X′ G G C T A G ^5′^	**5g**	7.0	10.4	85.2	14.8
^3′^ GTCCTCT X′ CTCTCC ^5′^	**5h**	5.7	11.1	86.3	13.7
^3′^ G T TG T A X′ A T GTT G AG ^5′^	**5j**	4.6	10.4	95.8	4.2
^3′^ G T T G TA X′ AT G T T GAG ^5′^	**5k**	3.5	10.4	96.7	3.3

^[a]^ DNA blue, LNA red, hydroxyprolinol with catalyst attached green. General conditions: 95 hours incubation at 37 °C; 50 mM Tris buffer pH 8.0, 100 mM NaCl; 150 nM conjugate **5**, 8.4–11.1‐fold excess of the target RNA.

The two fragments of RNA **8** resulting from the cleavage by conjugate **5k** were also separated by HPLC and characterized by their mass spectra. The faster running peak showed a deconvoluted mass of 6201.7 Da, corresponding to the sequence ^5′^UUACAACUCAUUCCAGTTTT. The second peak with a mass of 5588.9 Da contained the 2′,3′ cyclic phosphate of Cy5‐^5'^T_10‐_CAACAU (Figure S57). This confirmed the cleavage site assignment from Figure 7. Interestingly, the reaction did not form the thermodynamically most stable products, the 2′ and 3′ mono phosphates: fast dissociation of the fragment from the catalyst conjugate not only enabled substrate turnover but also prevented hydrolysis of the strained cyclic phosphate.

**Figure 8:**
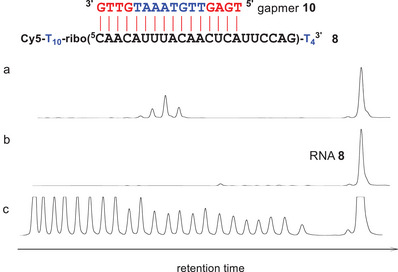
a) Cleavage of RNA **8** (150 nM) by RNase H (0.5 units) in the presence of gapmer **10** (360 nM, stereorandom phosphothioate linkages) after incubation for 2 minutes at 37 °C (50 mM Tris buffer pH 8.3, 75 mM KCl, 3 mM MgCl_2_, 10 mM DTT). Complete RNA cleavage occurred after extended incubation time. b) Same conditions as before but gapmer **10** was replaced by oligo **4k** lacking the catalyst (360 nM). The incubation time was extended from 2 minutes to 20 hours. c) Hydrolysis ladder of RNA **8**. In contrast to the 3′ phosphorylated fragments of the hydrolysis ladder, RNase H products are 5′ phosphorylated. Note that lanes a and c are not directly comparable. RNA is shown in black, DNA in blue, LNA in red.

Today, the most prevalent approach to achieve a site‐specific degradation of RNA is incubation with gapmers in the presence of RNase H.^[^ ^3b^ ^]^ RNA **8**, therefore, was treated with gapmer **10** and 0.5 units of the enzyme (Figure 8, lane a). Very short reaction times (2 minutes) were sufficient to cleave about 40% of the substrate. In contrast, the hybrid of RNA **8** and the DNA‐LNA mixmer **4k** was completely stable against RNase H: Only traces of fragments were formed, even after long incubation times (20 hours, Figure 8, lane b). The rate advantage of the gapmer/RNase H approach, however, is accompanied with a significantly lower level of precision: Four main RNA fragments were formed whereas conjugate **5k** cleaves RNA **8** in a single position.

The 412mer transcript **9** contains the sequence of model RNA **8** in positions 163–184 (for details see Supporting Information). Thus, conjugate **5k** should cleave the 412mer into fragments of 168 and 244 ribonucleotides. As a positive control, RNA **9** was also incubated with RNase H in the presence of gapmer **10**. Comparable fragment bands were formed in each of the reactions (Figures S58 and S59): apart from differences in kinetics, both methods can be used to cleave the 412mer RNA in a site‐specific way.

## 3 Conclusion

The attachment of benzimidazole catalysts to a central position of oligonucleotides by click reactions has several advantages. The synthesis starts from a stable catalyst azide **2** and a commercial alkyne linker **3**. In addition, the ligation step is less critical than the moisture sensitive coupling of phosphoramidites.^[^ ^12^ ^]^ Due to the fact that no subsequent deprotection is required, it should be possible to attach highly reactive but base sensitive analogs of **2** in the same way.^[^ ^9b^ ^]^ In contrast to 5′ conjugates of benzimidazole catalysts (Figure 1), compounds **5a**–**5k** in the present study all showed multiple substrate turnover combined with precisely defined cleavage sites. These conjugates, in particular **5k**, can be used for targeted RNA destruction. For gene silencing, however, the combination of gapmers and RNase H will remain the method of choice because benzimidazole catalysts are still rather slow. In terms of *k*_cat_ RNase H is superior by orders of magnitude.^[^ ^15^ ^]^ On the other hand, cleavage rates of **5k** (*k*_obs_ = 0.2 h^−1^) are in the same range as those of XNAzymes used to destroy viral RNA in cells (*k*_obs_ = 0.18–0.4 h^−1^).^[^ ^5e^ ^]^ Conjugates of type **5** also have specific advantages when high precision of RNA cleavage is required, e.g. for subsequent fragment analysis by mass spectrometry or similar techniques. When gapmer cleavage forms a bundle of related fragments (Figure 8), essentially a single product results from the reaction of **5k** (Figure 7). Although longer incubation is required, Mg^2+^‐free conditions help to reduce background cleavage. Conjugates **5** even work in the presence of EDTA. Thus, they may become useful tools for bioanalytical applications.^[^ ^12^, ^16^ ^]^

## Supporting Information

Experimental details, including the preparation of catalyst azide **2**, the synthesis of oligonucleotides **4** and of conjugates **5**. Characterization of conjugates **5** by HPLC and mass spectrometry. In‐line probing and RNA cleavage kinetics. Cleavage of 412mer RNA **9** by conjugate **5k** and by gapmer **10**.

## Conflict of Interests

The authors declare no conflicts of interest.

## Supporting information



Supporting information

## Data Availability

The data that support the findings of this study are available in the supplementary material of this article.
