# Supplementary material for: Click Conjugates of Artificial Ribonucleases: Sequence Specific Cleavage with Multiple Turnover
Source: Chemistry. 2025 May 6;31(30):e202500451. doi: 10.1002/chem.202500451 (PMC12117175; doi:10.1002/chem.202500451)
Supplement: Supplementary file 1 — Supporting information [file CHEM-31-e202500451-s001.pdf]

Click Conjugates of Artificial Ribonucleases: Specific Cleavage with Multiple Turnover

Sandra Weber, Timo Weinrich, Ute Scheffer, Elisabeth Kalden, and Michael W. Göbel*

Supporting Information

Table of Contents:

General information 2
Synthesis of catalyst **2** 3
Synthesis and purification of oligonucleotides 5
Click protocol 6
Preparation and analysis of cleavage experiments 6
^{1}H and ^{13}C NMR spectra 9
In-line probing experiments 13
Tm determination for the duplex **4a**·**6** 15
Saturation experiments 16
Determination of k_{obs} 21
Substrate specificity of conjugates 26
Temperature dependence of RNA cleavage by conjugate **5a** 28
HPLC chromatograms and mass spectra 29
Cleavage of 412mer transcript **9** 40

General information

Anhydrous solvents were purchased from Thermo Fisher Scientific. TLC plates were purchased from Macherey-Nagel (ALUGRAM Xtra SIL G UV254, aluminum sheets silica gel 60, 0.20 mm) and visualized by exposure to UV light or by staining with ninhydrin solution. Column chromatography: silica gel 60 (0.04 – 0.063 mm pore size). Proton nuclear magnetic resonance (^{1}H NMR) spectra and carbon nuclear magnetic resonance (^{13}C NMR) spectra were recorded at 300 K with Bruker AV 400 (^{1}H 400.3 MHz, ^{13}C 100.6 MHz) and AV 500 (^{1}H 500.2 MHz, ^{13}C 125.7 MHz) NMR spectrometers. The chemical shifts for protons and for carbon are reported in parts per million (ppm, δ scale) and internally referenced to the proton resonances of the solvent ($CDCl_3$: 7.26, DMSO-d_6: 2.50) and the carbon resonances of the solvent ($CDCl_3$: 77.16, DMSO-d_6: 39.52). Data are represented as follows: chemical shift [ppm], multiplicity (s = singlet, bs = broad singlet, d = doublet, dd = doublet of doublets, t = triplet, m = multiplet), coupling constants [Hz] and integration. ESI mass spectra: Thermo MSQ plus single quadrupole detector. Precision mass spectra: Bruker MicrOTOF-QII. Mass spectra of oligonucleotides **4** and **5**: LC-MS system from Thermo Fisher consisting of a Vanquish Flex pump and the Orbitrap Exploris 120 mass spectrometer. IR-spectra: Jasco FT/IR-420 spectrometer equipped with a diamond ATR unit.

Synthesis of catalyst 2

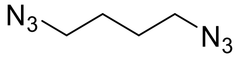

S1

1,4-Diazidobutane S1. To 1,4-dibromobutane (4.00 g, 18.5 mmol, 1.0 eq.), dissolved in 90 mL DMF abs. and 10 mL H_2O, sodium azide (3.01 g, 46.3 mmol, 2.5 eq.) was added. The mixture was stirred vigorously and heated to 75 °C under inert atmosphere overnight. The solution was extracted with Et_2O and the combined organic layers were washed with H_2O and saturated NaCl solution. The organic phase was dried over $MgSO_4$ and the solvent was in part removed under reduced pressure. Do not evaporate to dryness due to potential explosiveness of the product! A fraction of the desired product was obtained as a colorless oil (extrapolated yield: 1.88 g, 72 %).

^{1}H NMR (400.3 MHz, $CDCl_3$): δ [ppm] = 3.35 – 3.30 (m, 4 H), 1.71 – 1.64 (m, 4 H).

^{13}C NMR (100.6 MHz, $CDCl_3$): δ [ppm] = 51.04, 26.28.

IR: v [cm^{-1}] = 2939 (w), 2084 (vs), 1724 (m), 1452 (w), 1350 (w), 1254 (b), 1171 (m), 1011 (w), 893 (w), 744 (w), 631 (w), 619 (w).

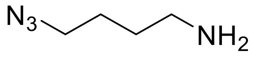

S2

4-Azidobutylamine S2. Compound **S1** (1.84 g, 13.1 mmol, 1.0 eq.) was dissolved in a mixture of 20 mL Et_2O, 20 mL EtOAc and 30 mL 1M HCl and cooled to 0 °C under inert atmosphere. While stirring vigorously, PPh_3 (3.43 g, 13.1 mmol, 1.0 eq.) was added in portions. The mixture was stirred overnight at ambient temperature. Phases were separated and the aqueous phase was washed with CH_2Cl_2 to get rid of $OPPh_3$. Afterwards, the aq. phase was basified by adding a 15 % NaOH solution and extracted with CH_2Cl_2. The combined organic layers were dried over $MgSO_4$ and the solvent was removed under reduced pressure. Due to the high volatility of the product, the solvent should not be removed completely under reduced pressure. The crude product (yellowish oil) contained some CH_2Cl_2 and the yield (1.40 g, 93 %) was calculated based on the ratio product/solvent according to ^{1}H-NMR.

R_f = 0.64 (CH_2Cl_2/MeOH 5/1, 1 % aq. NH_3)

^{1}H NMR (400.3 MHz, $CDCl_3$): δ [ppm] = 3.29 (t, *J* = 6.8 Hz, 2 H), 2.73 (t, *J* = 6.9 Hz, 2 H), 1.68 – 1.61 (m, 2 H), 1.56 – 1.49 (m, 2 H), 1.36 (s, 2 H).

^{13}C NMR (100.6 MHz, $CDCl_3$): δ [ppm] = 51.49, 41.82, 30.90, 26.44.

MS (ESI) m/z: 115.08 $[M+H]^+$, calc. for $C_4H_{11}N_4^+$ 115.16

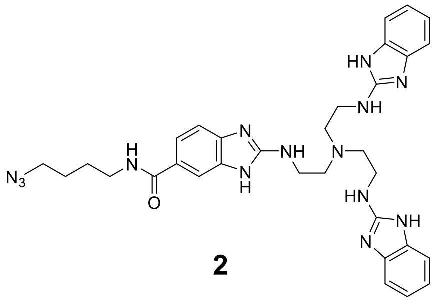

2

***N*-(4-Azidobutyl)-2-((2-(bis(2-((1*H*-benzo[*d*]imidazol-2-yl)amino)ethyl)amino)-ethyl)amino)-1*H*-benzo[*d*]imidazole-6-carboxamide 2.** Tris(2-aminobenzimidazo-le)carboxylic acid **1** (1.00 g, 1.86 mmol, 1.0 eq.) was dissolved in 20 mL dry DMF under argon and DIPEA (0.97 mL, 5.56 mmol, 3.0 eq.), HOBt (0.56g, 3.65 mmol, 2.0 eq.) and DIC (0.60 mL, 3.65 mmol, 2.0 eq.) were added. After 30 min amine **S2** (0.42 g, 3.65 mmol, 2.0 eq.) was added and the reaction mixture was stirred overnight at ambient temperature. Saturated $NaHCO_3$ solution was added and the aqueous phase was extracted with EtOAc. The combined organic layers were dried over $MgSO_4$ and the solvent was removed under reduced pressure. The residue was purified by column chromatography (SiO_2, CH_2Cl_2/MeOH 9/1, 1 % aq. NH_3) to obtain the desired product **2** as a beige solid (200 mg, 17 %).

R_f = 0.38 (CH_2Cl_2/MeOH 7/1, 1 % aq. NH_3)

^{1}H NMR (500.2 MHz, DMSO-d_6): δ [ppm] = 8.23 (t, *J* = 5.7 Hz, 1H), 7.65 (d, *J* = 1.7 Hz, 1H), 7.45 (dd, *J* = 8.2, 1.7 Hz, 1H), 7.14 – 7.10 (m, 5H), 6.90 – 6.86 (m, 4H), 6.84 (bs, 1H), 3.43 – 3.40 (m, 6H), 3.37 – 3.34 (m, 2H), 3.27 – 3.24 (m, 2H), 2.79 (t, *J* = 6.6 Hz, 6H), 1.58 – 1.55 (m, 4H).

^{13}C NMR (125.7 MHz, DMSO-d_6): δ [ppm] = 167.03, 156.85, 155.19, 125.74, 119.48, 111.52, 53.55, 50.43, 40.56, 38.52, 26.55, 25.88.

MS (ESI) m/z: 635.26 $[M+H]^+$, calc. for $C_{32}H_{39}N_{14}O^+$ 635.34

HRMS (ESI) m/z: 635.3404 $[M+H]^+$, calc. for $C_{32}H_{39}N_{14}O^+$ 635.3426

Synthesis and purification of oligonucleotides

General. DNA synthesis was executed on an Expedite Nucleic Acid Synthesizer from PerSeptive Biosystems. RP-HPLC was carried out on a Jasco LC-900 HPLC system with a UV-975 UV/VIS detector. Column: Phenomenex Jupiter 4 µm Proteo 90 Å (250 × 10 mm). All samples were concentrated in a SpeedVac and diluted in autoclaved DEPC-H_2O.

Synthesis. Oligonucleotide synthesis was done using a standard coupling protocol at 1.0 µmol scale. Trichloroacetic acid in CH_2Cl_2 (deblock solution), acetic anhydride in THF (Cap A), *N*-methylimidazole in THF/pyridine (Cap B) and iodine in pyridine/H_2O/THF (oxidizer) were purchased from SAFC Proligo (Sigma Aldrich). 0.3 M 5-benzylmercaptotetrazole in acetonitrile (activator) was acquired from emp BIOTECH. DNA and LNA amidites and packed CPG columns were purchased from Biosearch Technologies. Hydroxyprolinol amidite **3**: Lumiprobe. Dry acetonitrile for dilution of the amidites was purchased from Sigma Aldrich. All oligonucleotides were synthesized "Trityl-On".

Isolation and purification. For cleavage from solid support and deprotection of the oligonucleotides **4**, the CPG was removed from the column and incubated with 1 mL of 32% aq. ammonia/EtOH (3/1) at 37 °C for 20 h in an Eppendorf tube. Afterwards, the supernatant was separated from the CPG support and the ammonia was removed under reduced pressure. The purification was performed by RP-HPLC. RP conditions: A: MeOH B: aqueous HFIP (400 mM)/Et_3N (16 mM) pH 7.9; gradient: 5% A and 95 % B 0 – 2 min, 5 % to 69 % A and 95 % to 31 % B from 2 – 22 min. The column was heated to 60 °C. After evaporating the oligonucleotide to dryness, it was incubated with 300 µL of acetic acid for 30 minutes at room temperature, to cleave off the 5'OH-Trityl group. This was followed by another RP-HPLC purification (same conditions as above) after evaporation of the acetic acid.

Quantification. Concentrations of the oligonucleotides were determined with a nanodrop2000 *via* UV spectrometry using Lambert Beers law. Extinction coefficients were calculated by a nearest neighbor model.

Click protocol

Urea (170 mg, 2.83 mmol) is dissolved with 245 µL 0.1 M TEAA – buffer (pH 7.0) in a 2 mL Eppendorf tube. The oligonucleotide **4** (15 µL, 15 nmol, 1.0 eq., 1.00 mM) is added and the solution is mixed briefly. Thereafter, a premixed solution of $CuSO_4$/TBTA (7.5 µL, 6.67 mM/33.33 mM) is added and the reaction mixture is slightly vortexed again. In addition, a solution of catalyst azide **2** (40 µL, 200 nmol, 13.0 eq., 5.00 mM) and a freshly prepared sodium ascorbate solution (25 µL, 2500 nmol, 167 eq., 100 mM) are added. Afterwards the Eppendorf tube is flushed with argon and the solution is mixed again. The reaction takes place for 4 days at room temperature and is vortexed several times. The desired product is isolated by RP – HPLC. (Same conditions as described before). Depending on sequence, between 30 and 70 % of oligonucleotides **4** are converted into conjugates **5**.

$CuSO_4$/TBTA (1:5): 2.5 µL 20 mM $CuSO_4$ in DEPC-H_2O + 5 µL 50 mM TBTA in DMSO

reagent	solvent	end concentration	equivalents
oligonucleotide	DEPC-H_2O	45 µM	1.0
$CuSO_4$	DEPC-H_2O	150 µM	3.3
TBTA	DMSO	750 µM	16.5
catalyst azide **2**	DMSO	600 µM	13.0
Na ascorbate	DEPC-H_2O	7.50 mM	167.0
urea	DEPC-H_2O	8.50 M	-----

Preparation and analysis of cleavage experiments

General. *In-line* probing experiments, saturation and kinetic measurements were carried out in 1.5 mL DNA LowBind microtubes (Sarstedt). Samples were quenched with urea loading buffer (stock: 8.0 M urea, 20 mM EDTA, 0.2 % crocein orange). DNA and RNA oligonucleotides were dissolved in DEPC-H_2O. Analysis of the cleavage experiments was carried out by gel electrophoresis (16 % polyacrylamide, 7 M urea) on a Pharmacia ALFexpress II sequencer; gels prepared from Rotiphorese Gel 40 (19:1) stock solution from Carl Roth and 10xTBE buffer. RNase H and RNase H buffer were purchased from New England Biolabs. Conditions of gel electrophoresis: 350 min, 1500 V, 60 mA, 25 W, 57 °C.

In-line Probing. 4 µL DEPC-H_2O, 2 µL *in-line* buffer pH 8.3 (stock: 250 mM Tris-HCl, 100 mM $MgCl_2$, 500 mM KCl), 2 µL oligonucleotide **4** (7.5 µM), 2 µL RNA substrate (750 nM). The solution was vortexed gently and centrifuged after adding each

compound. The microtube was incubated for 20 h at 37 °C. The reaction was stopped by adding 15 µL of urea loading buffer. The mixture was vortexed and centrifuged and 10 µL of this solution were used for gel electrophoresis.

Saturation experiments. 4 µL DEPC-H_2O, 2 µL Tris-buffer pH 8.0 (stock: 250 mM Tris - HCl, 500 mM NaCl in DEPC-H_2O), 2 µL conjugate (0.75 µM - 11.25 µM), 2 µL substrate (750 nM). The solution was vortexed gently and centrifuged after adding each compound. The micro tube was incubated for 20 h at 37 °C. The reaction was stopped by adding 15 µL of urea loading buffer. The mixture was vortexed and centrifuged and 1 µL of this solution was used for gel electrophoresis.

Kinetic measurements. A stock solution was prepared by adding 24 µL of DEPC-H_2O, 12 µL of Tris-buffer pH 8.0 (stock: 250 mM Tris - HCl, 500 mM NaCl), and 12 µL of conjugate (concentration depending on RNA affinity, between 1875 nM to 7.5 µM). The solution was vortexed gently and centrifuged after adding each compound. 16 µL were taken from this stock solution and 4 µL of RNA substrate (750 nM) were added. The micro tube was incubated at 37 °C. At different times (1 h, 3 h, 5 h, 7 h, 10 h) 2 µL were taken from this solution, quenched with 3 µL of urea loading buffer and the samples were frozen in liquid nitrogen. After 10 h the samples were thawed and 1 µL of the solutions was used for gel electrophoresis.

Control experiments. 6 µL DEPC-H_2O, 2 µL Tris-buffer pH 8.0 (stock: 250 mM Tris, 1500 mM NaCl in DEPC-H_2O), 2 µL substrate (750 nM). The solution was vortexed gently and centrifuged after adding each compound. The micro tube was incubated for 20 h at 37 °C. The reaction was stopped by adding 15 µL of urea loading buffer. The mixture was vortexed and centrifuged and 10 µL of this solution were used for gel electrophoresis.

RNase H experiments. 4 µL of DEPC-H_2O, 1 µL of 10x RNase H buffer pH 8.3 (stock: 500 mM Tris-HCl, 750 mM KCl, 30 mM $MgCl_2$, 100 mM DTT), 2 µL of gapmer **10** or oligonucleotide **4k** (1.8 µM), 2 µL of RNA substrate (750 nM), 1 µL of RNase H (0.5 U). The solution was vortexed gently and centrifuged after adding each compound. After RNase H was added, the solution was just shaken and centrifuged to avoid adsorption of the enzyme on the surface of the microtube. The samples were incubated at 37 °C for 2 minutes (gapmer) and for 20 h (oligonucleotide **4k**). The reaction was quenched by adding 15 µL of urea loading buffer and 1 µL of this solution was used for gel electrophoresis.

Cleavage of RNA substrate 412mer 9. 4 µL of DEPC-H_2O, 1 µL of 10x RNase H buffer pH 8.3 (stock: 500 mM Tris-HCl, 750 mM KCl, 30 mM $MgCl_2$, 100 mM DTT), 2 µL of gapmer **10** (15 µM) or conjugate **5k** (15 µM), 2 µL of RNA substrate (10 µM), 1 µL of RNase H (0.5 U). The solution was vortexed gently and centrifuged after adding each compound. After RNase H was added, the solution was just shaken and centrifuged to avoid adsorption of the enzyme on the surface of the microtube. The samples were incubated at 37 °C for 3 minutes (gapmer **10**) and for 4.5 h (conjugate **5k**). The reactions were stopped by adding 10 µL of urea loading buffer. Afterwards the samples were incubated at 90 °C for 10 minutes and 10 µL of the solutions were added to the SDS page (8 % acrylamide, 7.0 M urea).

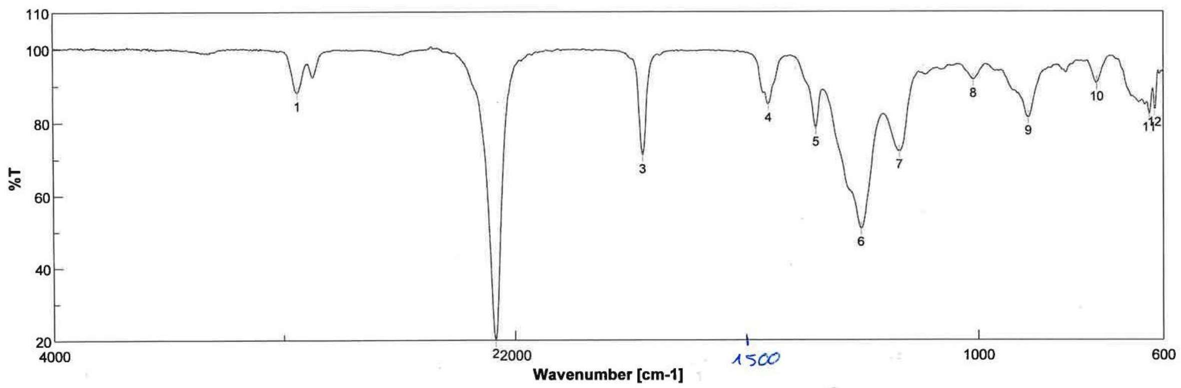

Accumulation	16	Resolution	4 cm-1
Zero Filling	OFF	Apodization	Cosine
Gain	8	Scanning Speed	2 mm/sec
Date/Time	20.11.2024 12:35	Update	20.11.2024 12:35
Operator			
File Name	Memory#5		
Sample Name			
Comment			

No.	cm-1	%T	No.	cm-1	%T	No.	cm-1	%T	No.	cm-1	%T	No.	cm-1	%T
1	2938.98	88.0426	2	2084.67	20.0454	3	1724.05	70.9534	4	1452.14	84.98	5	1349.93	78.3503
6	1253.5	50.772	7	1170.58	72.1622	8	1010.52	91.5561	9	892.88	81.3575	10	744.388	90.6126
11	630.609	82.2646	12	619.038	83.6281									

Figure S1: IR spectrum of 1,4-diazidobutane **S1**.

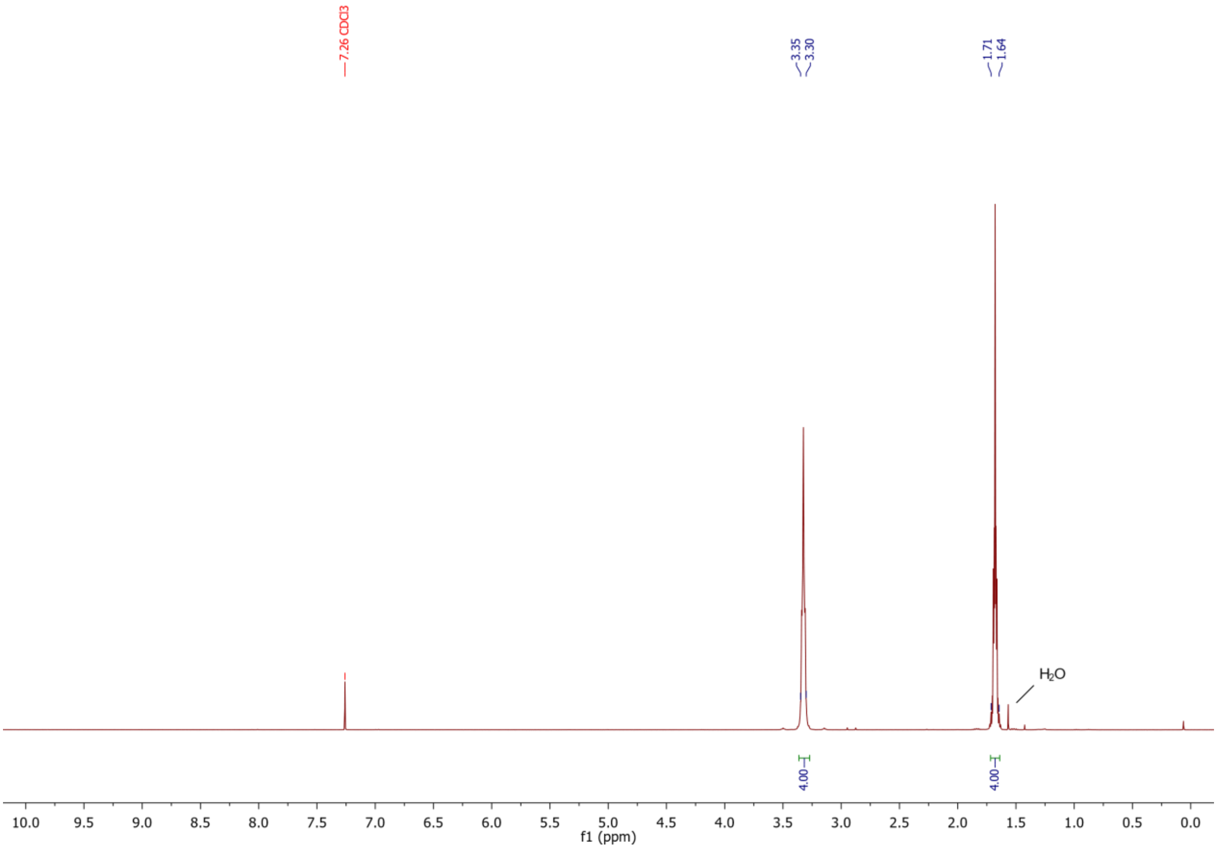

Figure S2: ^{1}H-NMR spectrum of 1,4-diazidobutane **S1**.

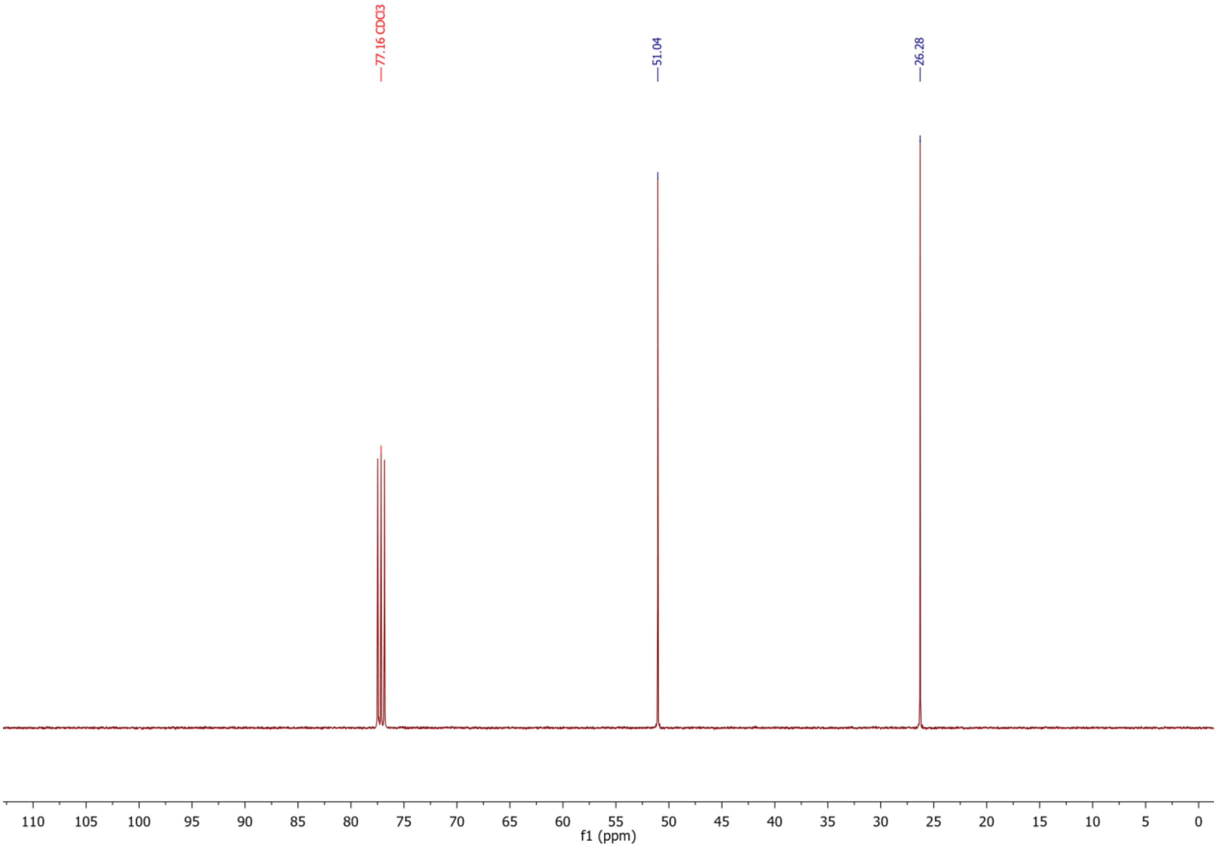

Figure S3: ^{13}C-NMR spectrum of 1,4-diazidobutane **S1**.

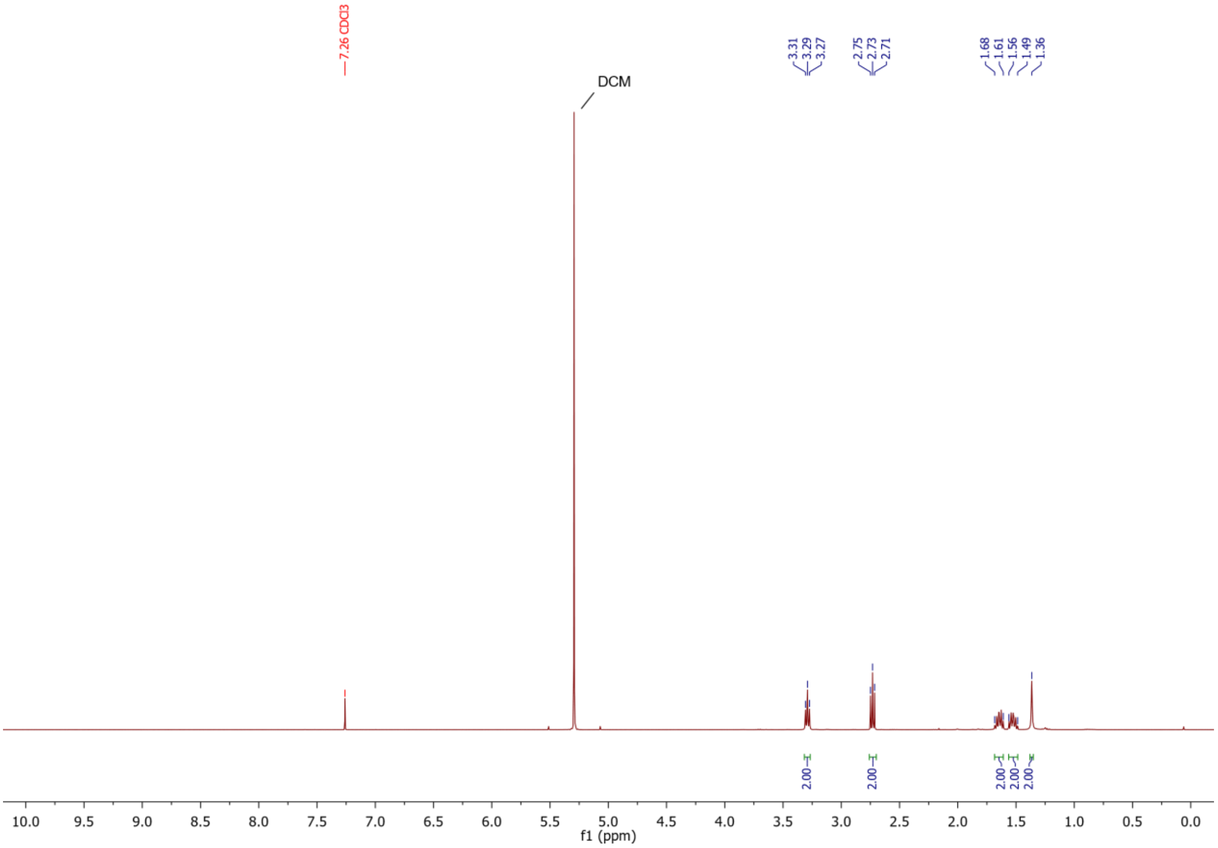

Figure S4: ^{1}H-NMR spectrum of 4-azidobutane-1-amine **S2**.

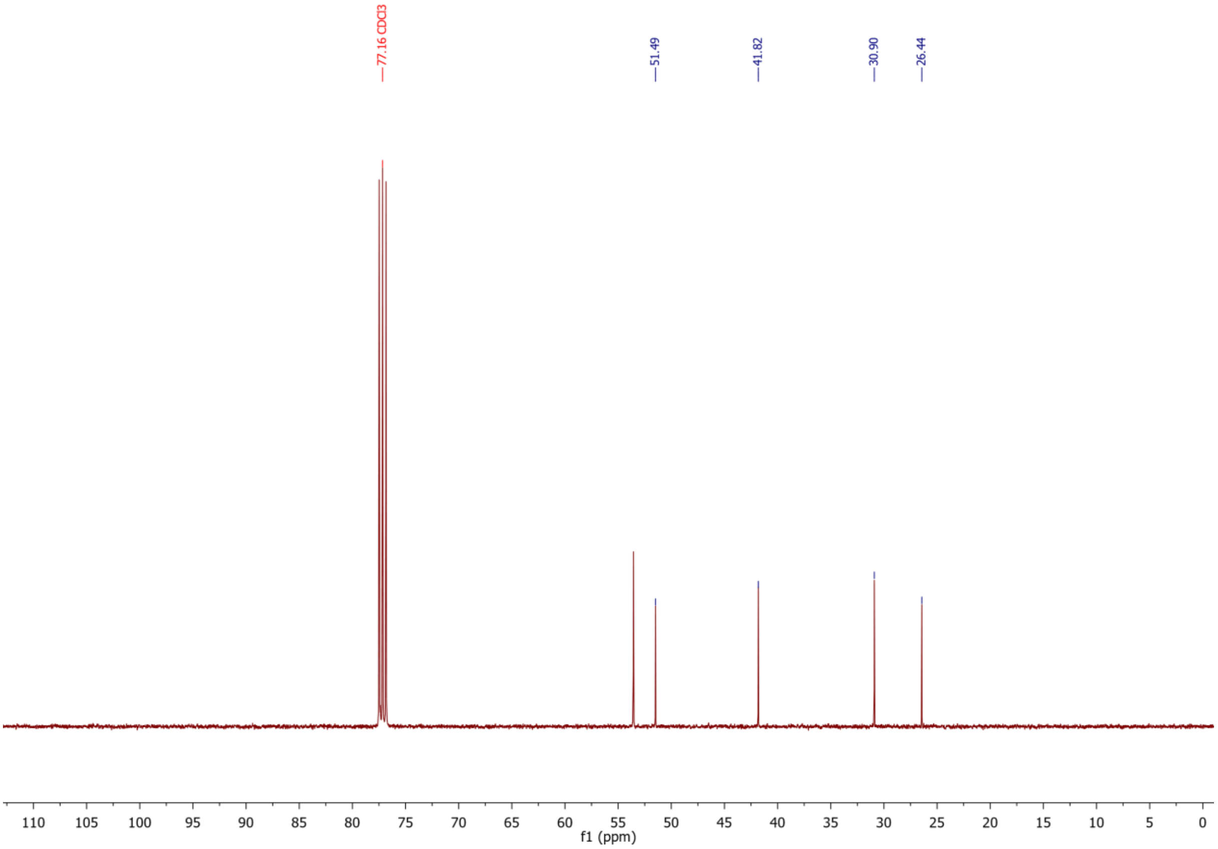

Figure S5: ^{13}C-NMR spectrum of 4-azidobutane-1-amine **S2**.

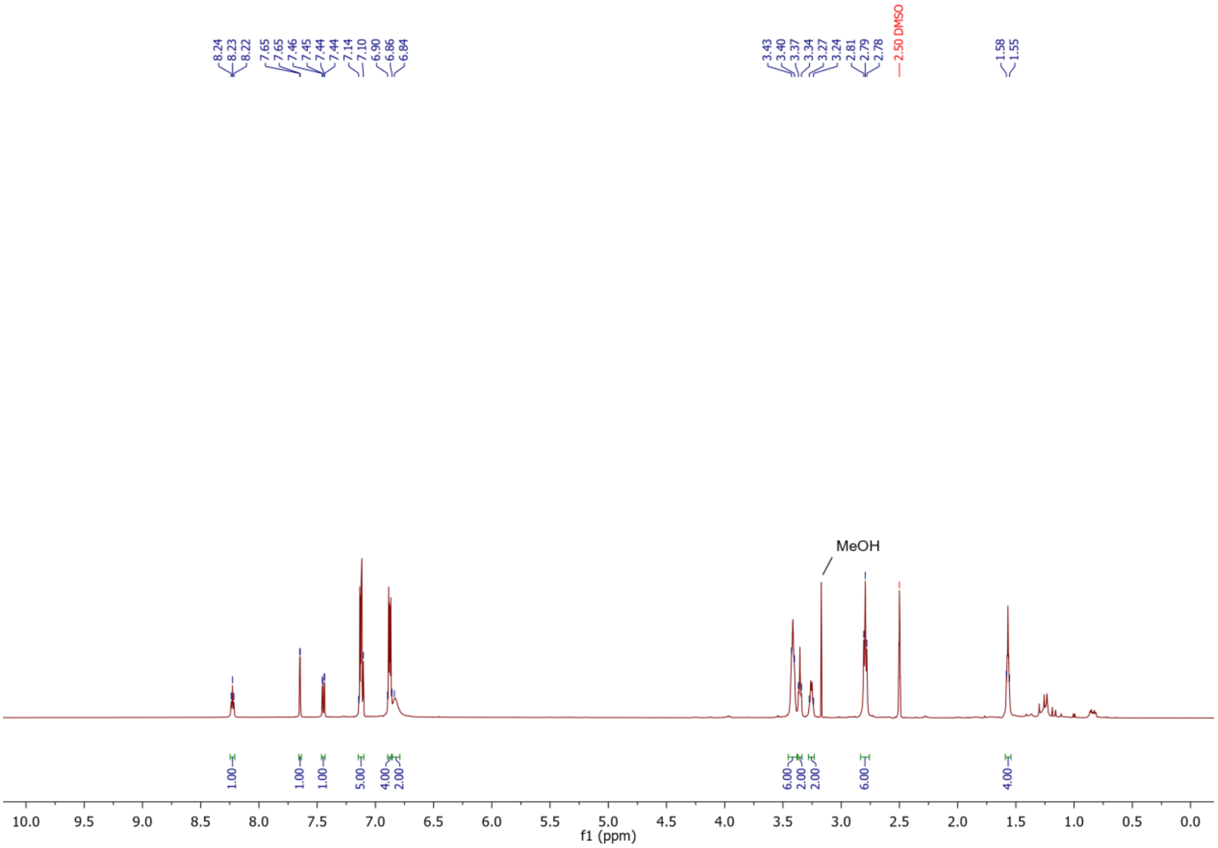

Figure S6: ^{1}H-NMR spectrum of catalyst azide **2**.

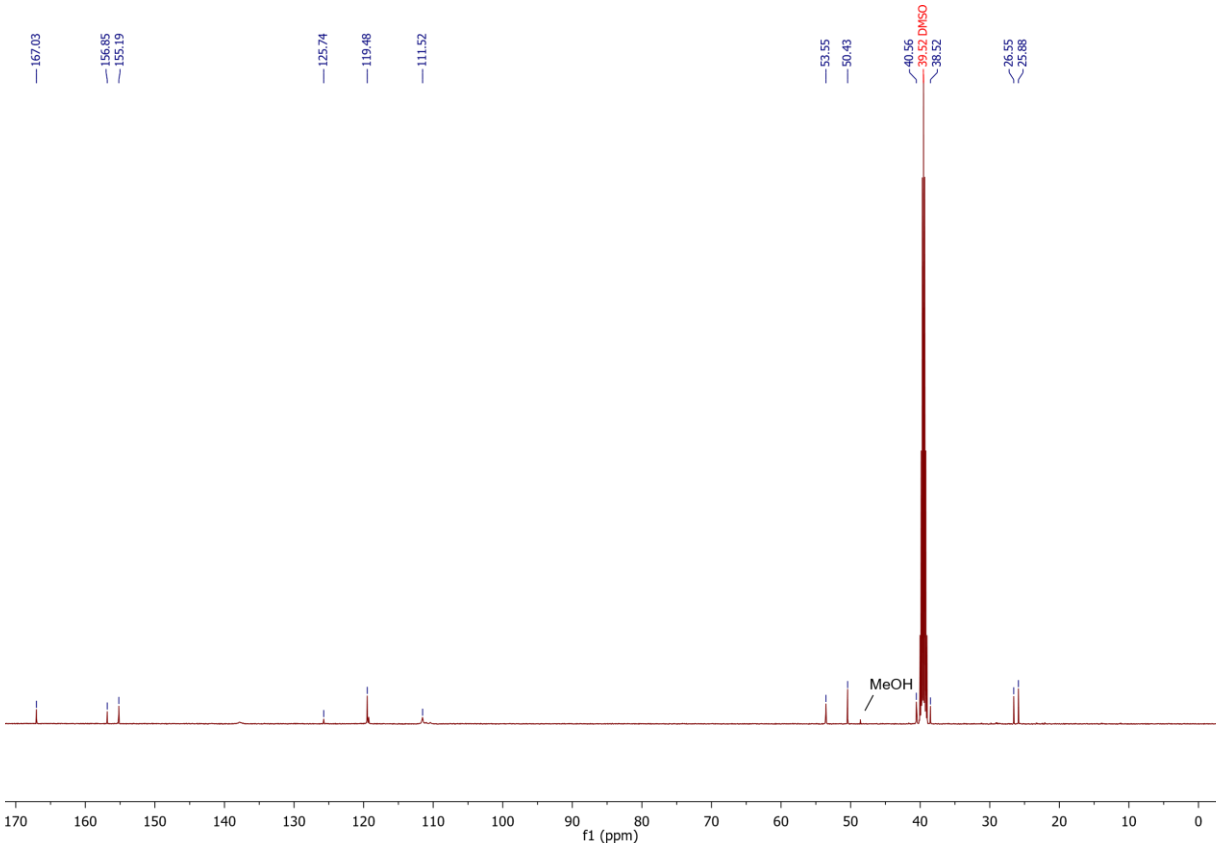

Figure S7: ^{13}C-NMR spectrum of catalyst azide **2**.

In-line probing experiments

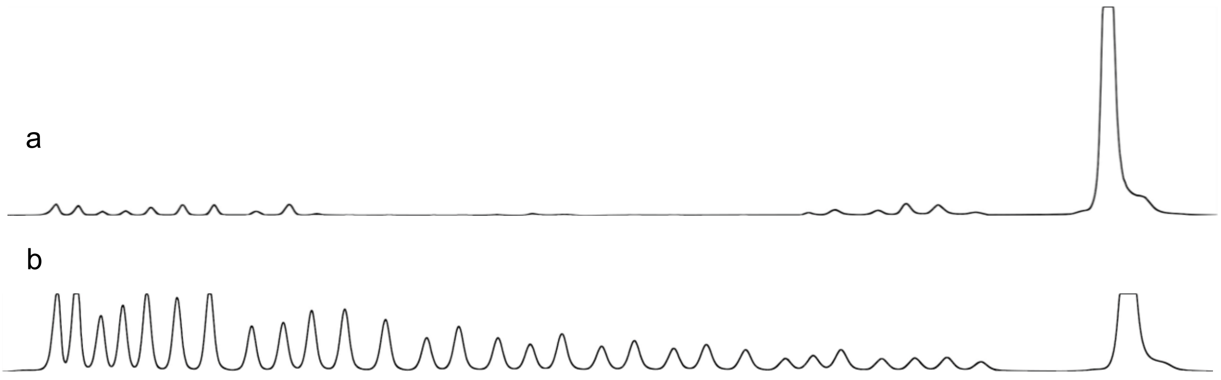

Figure S8: **a** In-line probing of conjugate **4h** (1.5 µM) and RNA **7** (150 nM), 50 mM Tris buffer (pH 8.3), 20 mM $MgCl_2$, 100 mM KCl, 37°C, 20 h. **b** hydrolysis ladder of RNA **7**.

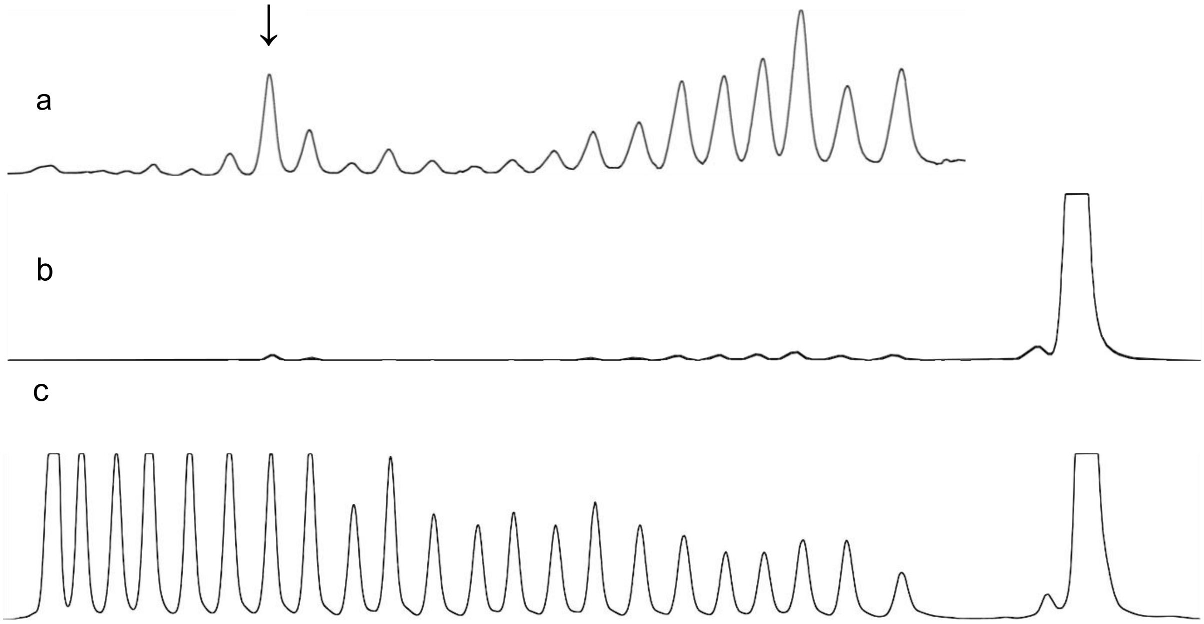

Figure S9: **a** Enlargement of lane b. The arrow indicates U(7). **b** In-line probing of oligo **4k** (1.5 µM) and RNA **8** (150 nM), 50 mM Tris buffer (pH 8.3), 20 mM $MgCl_2$, 100 mM KCl, 37°C, 20 h. **c** hydrolysis ladder of RNA **8**.

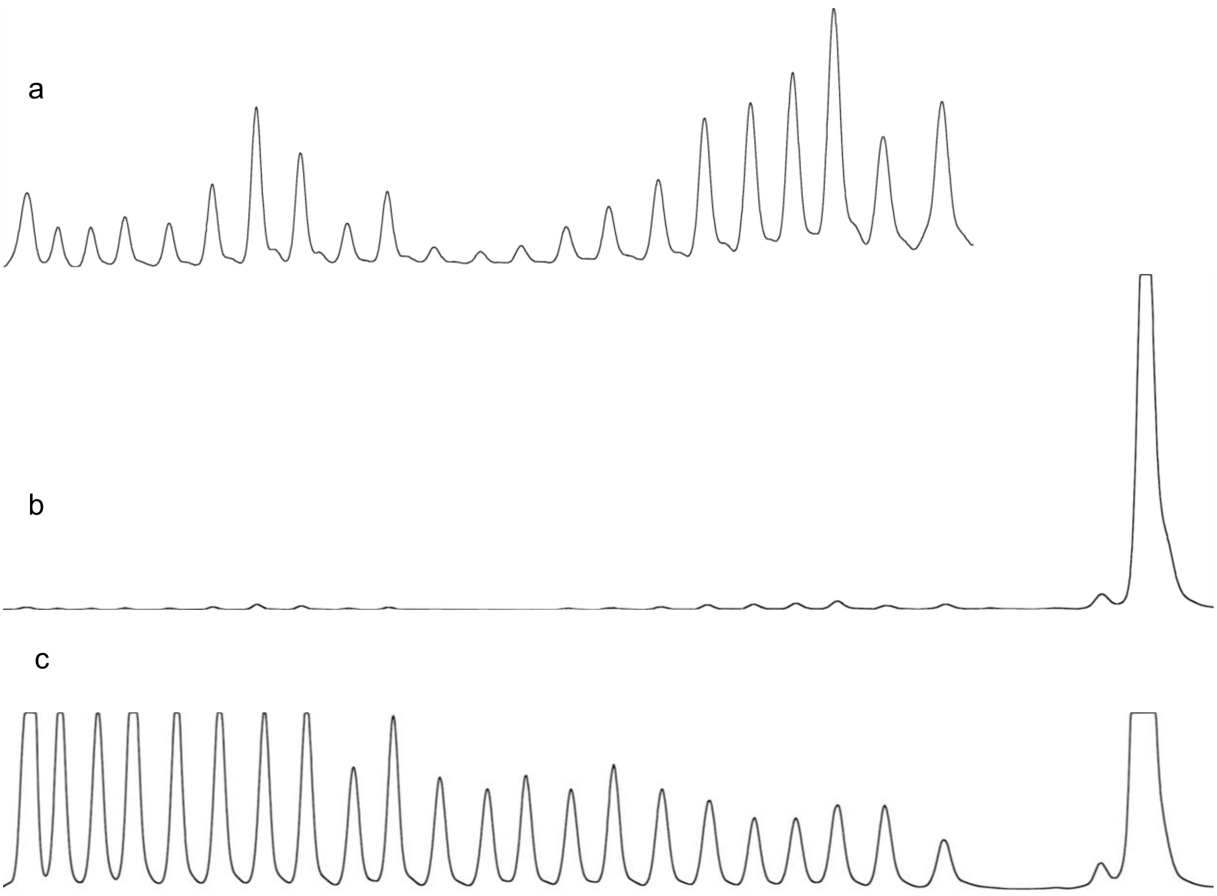

Figure S10: **a** Enlargement of lane b. **b** In-line probing of oligo **4i** (1.5 µM) and RNA **8** (150 nM), 50 mM Tris buffer (pH 8.3), 20 mM $MgCl_2$, 100 mM KCl, 37°C, 20 h. **c** hydrolysis ladder of RNA **8**.

Tm determination for the duplex 4a·6

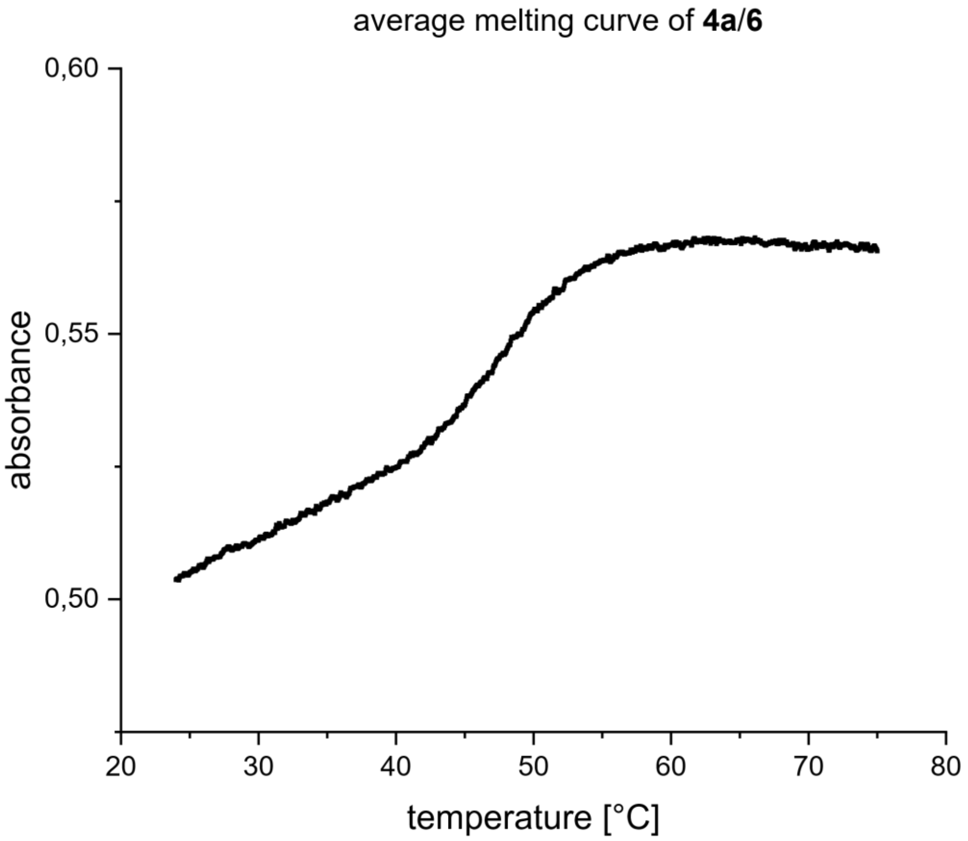

Figure S11: Plot of the average melting curve of oligo **4a** (1 µM) and substrate 6 (1 µM) in 1x PBS-buffer pH 7.4.

The determination of the melting temperature was performed on a UV-VIS spectrometer "Evolution 300" from Thermo Fisher Scientific equipped with a peltier element. Oligo **4a** (1 nmol) and substrate **6** (1 nmol) were mixed in a total volume of 1 mL 1x PBS-buffer pH 7.4 and the spectra were recorded in a 10.00 mm path length quartz glass cuvette from Hellma Analytics. For each cycle the sample was heated from 24 °C to 75 °C with a slope of 1 °C per minute and then cooled down to 24 °C again. The average melting point (point of inflection) was determined by using the data of 4 cycles: 47.0 ± 1.5 °C.

Saturation experiments

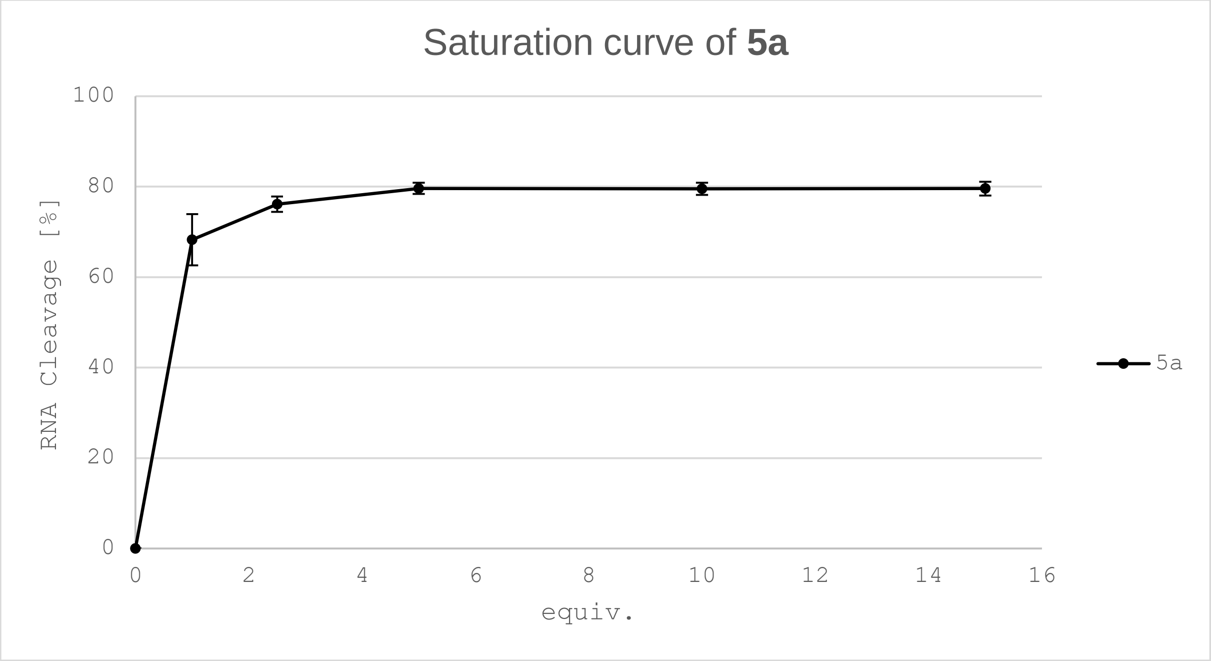

Figure S12: Saturation curve of conjugate **5a**. Conjugate **5a** (150 nM – 2.25 µM), RNA **6** (150 nM), 50 mM Tris-HCl pH 8.0, 100 mM NaCl, 37 °C, 20h.

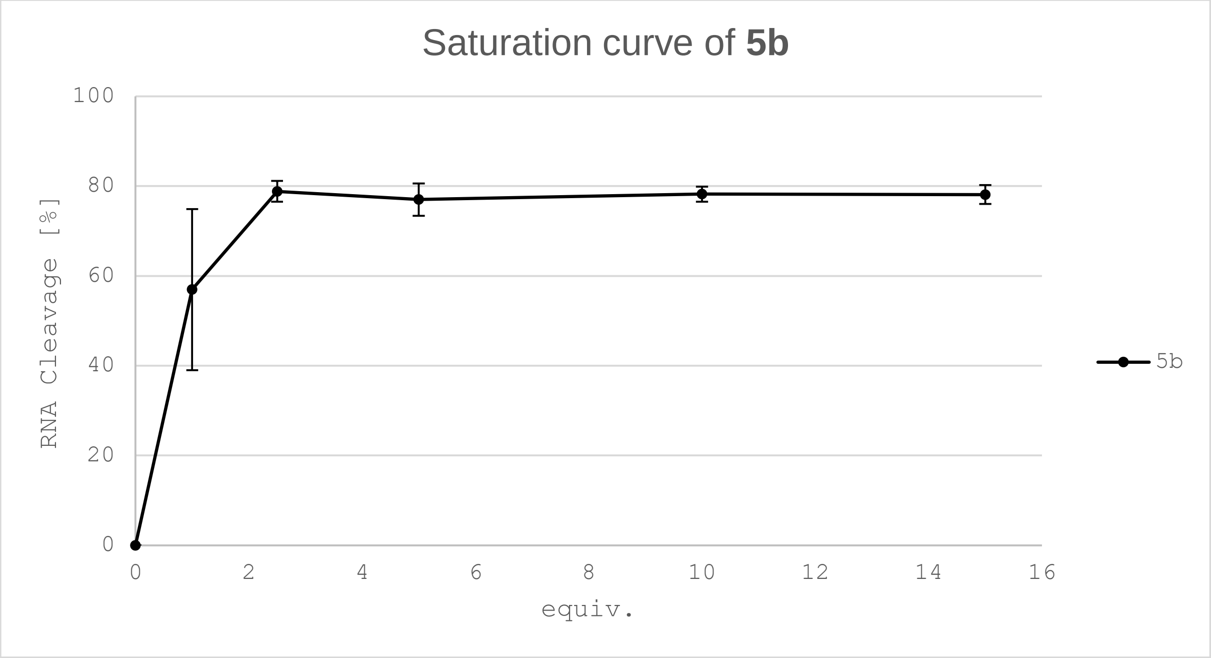

Figure S13: Saturation curve of conjugate **5b**. Conjugate **5b** (150 nM – 2.25 µM), RNA **6** (150 nM), 50 mM Tris-HCl pH 8.0, 100 mM NaCl, 37 °C, 20h.

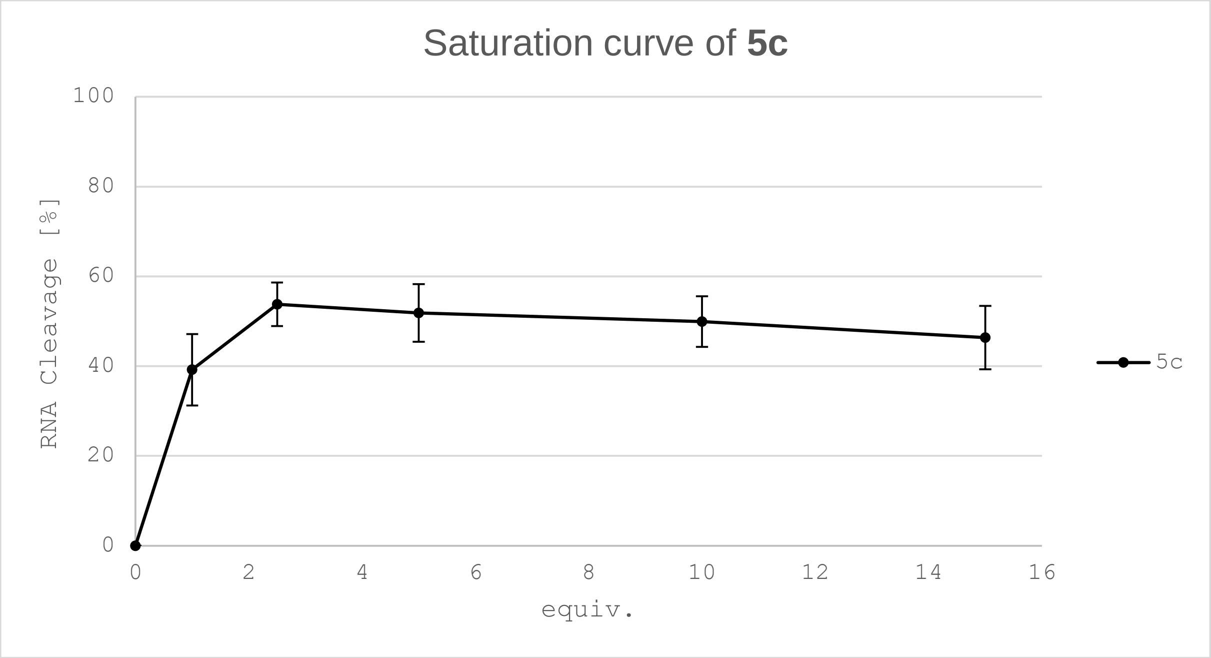

Figure S14: Saturation curve of conjugate **5c**. Conjugate **5c** (150 nM – 2.25 μM), RNA **6** (150 nM), 50 mM Tris-HCl pH 8.0, 100 mM NaCl, 37 °C, 20h.

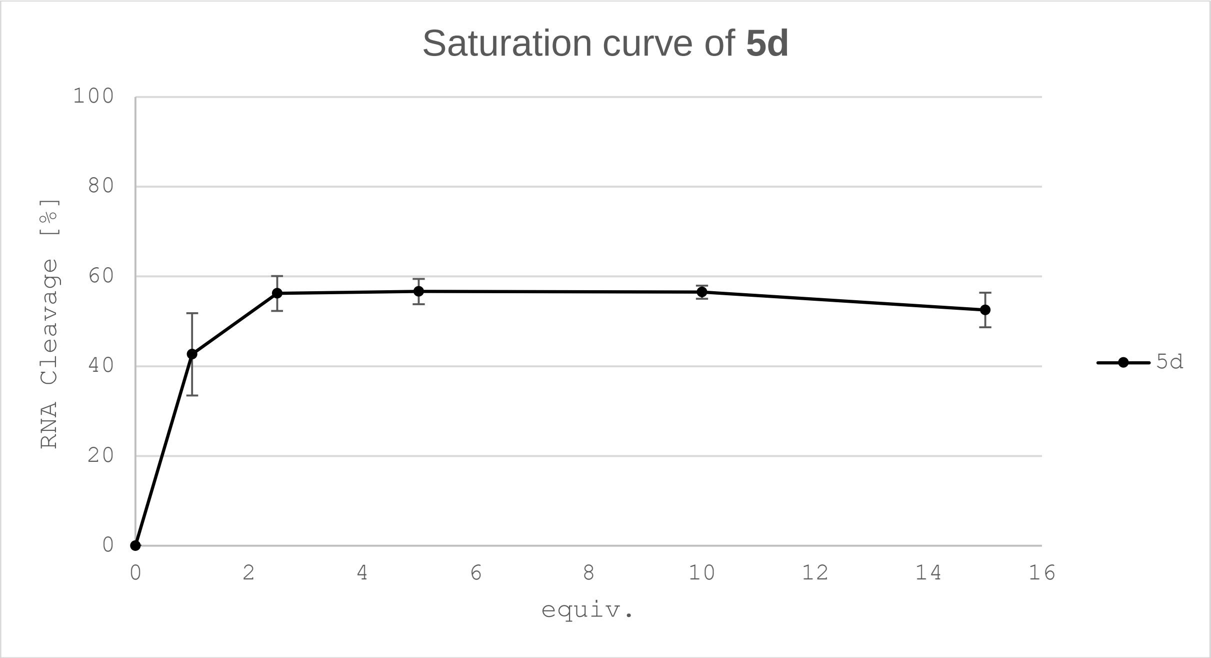

Figure S15: Saturation curve of conjugate **5d**. Conjugate **5d** (150 nM – 2.25 μM), RNA **6** (150 nM), 50 mM Tris-HCl pH 8.0, 100 mM NaCl, 37 °C, 20h.

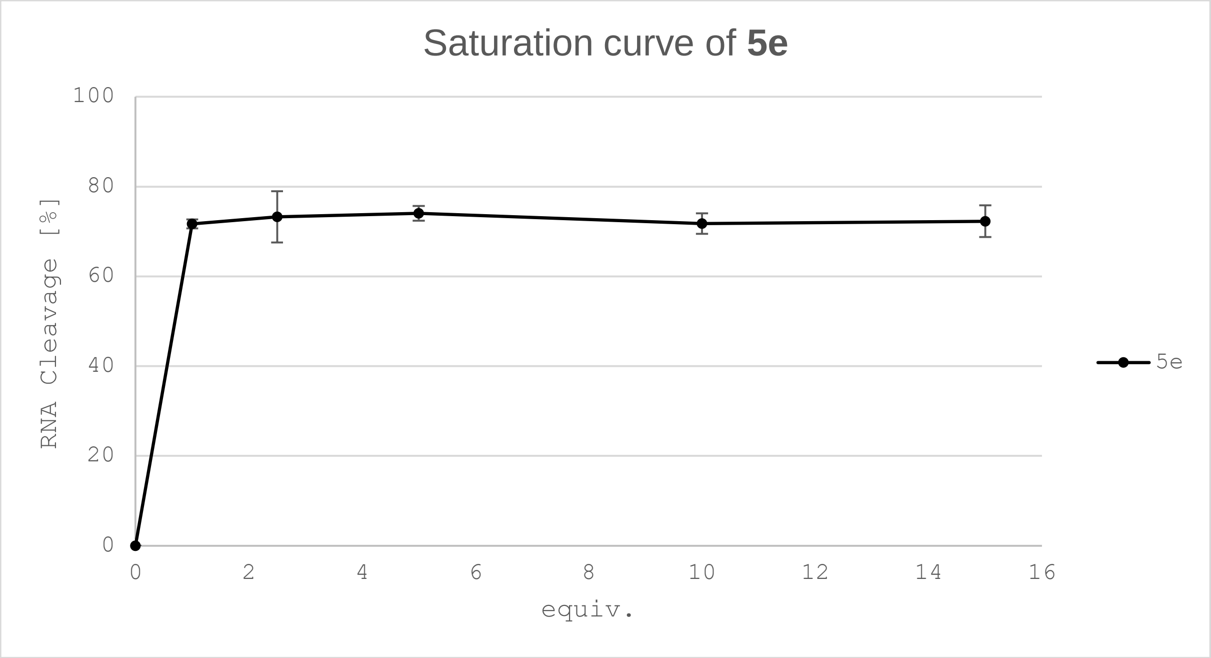

Figure S16: Saturation curve of conjugate **5e**. Conjugate **5e** (150 nM – 2.25 μM), RNA **6** (150 nM), 50 mM Tris-HCl pH 8.0, 100 mM NaCl, 37 °C, 20h.

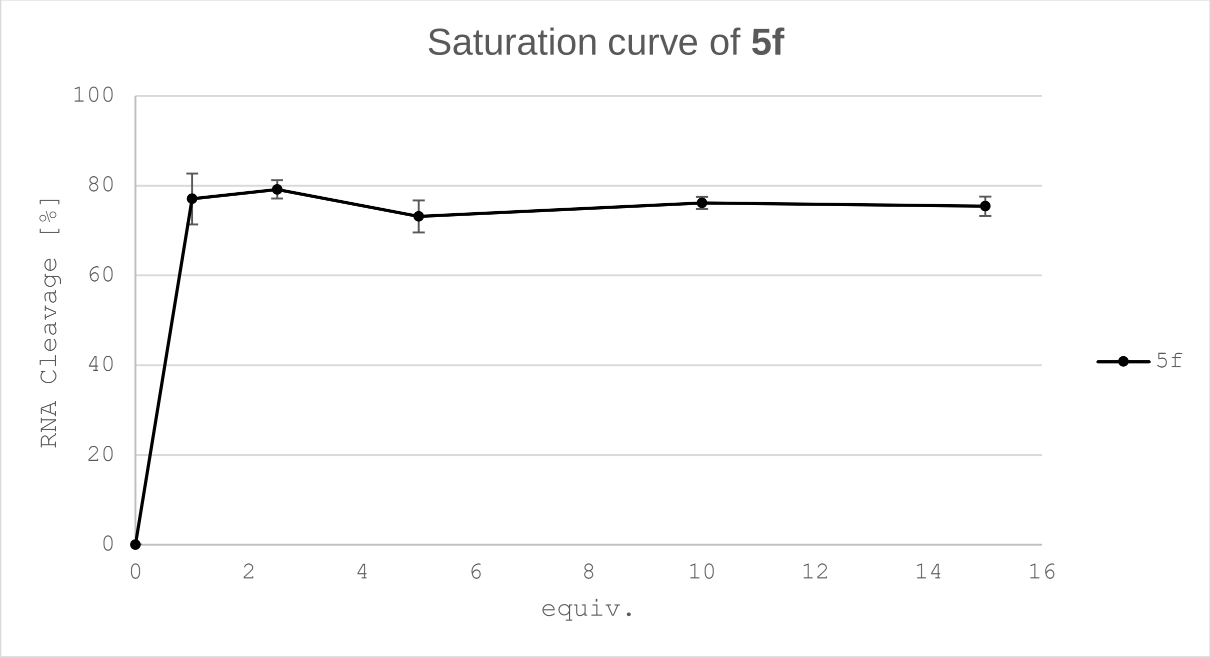

Figure S17: Saturation curve of conjugate **5f**. Conjugate **5f** (150 nM – 2.25 μM), RNA **6** (150 nM), 50 mM Tris-HCl pH 8.0, 100 mM NaCl, 37 °C, 20h.

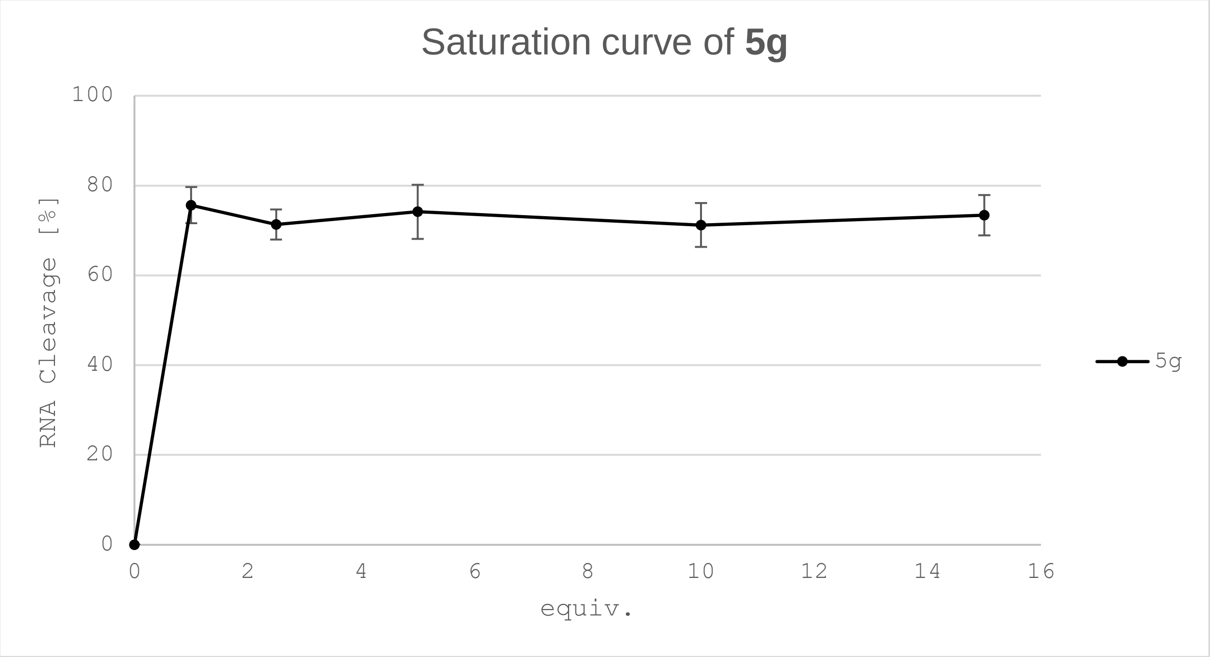

Figure S18: Saturation curve of conjugate **5g**. Conjugate **5g** (150 nM – 2.25 µM), RNA **6** (150 nM), 50 mM Tris-HCl pH 8.0, 100 mM NaCl, 37 °C, 20h.

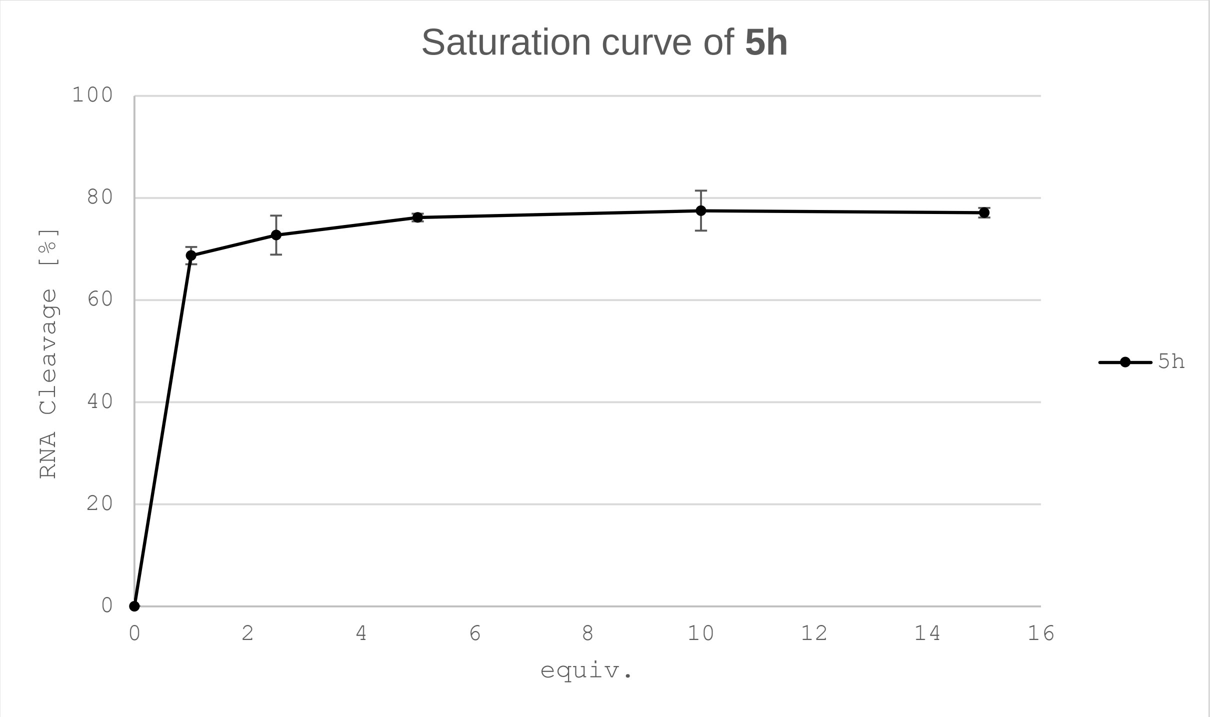

Figure S19: Saturation curve of conjugate **5h**. Conjugate **5h** (150 nM – 2.25 µM), RNA **7** (150 nM), 50 mM Tris-HCl pH 8.0, 100 mM NaCl, 37 °C, 20h.

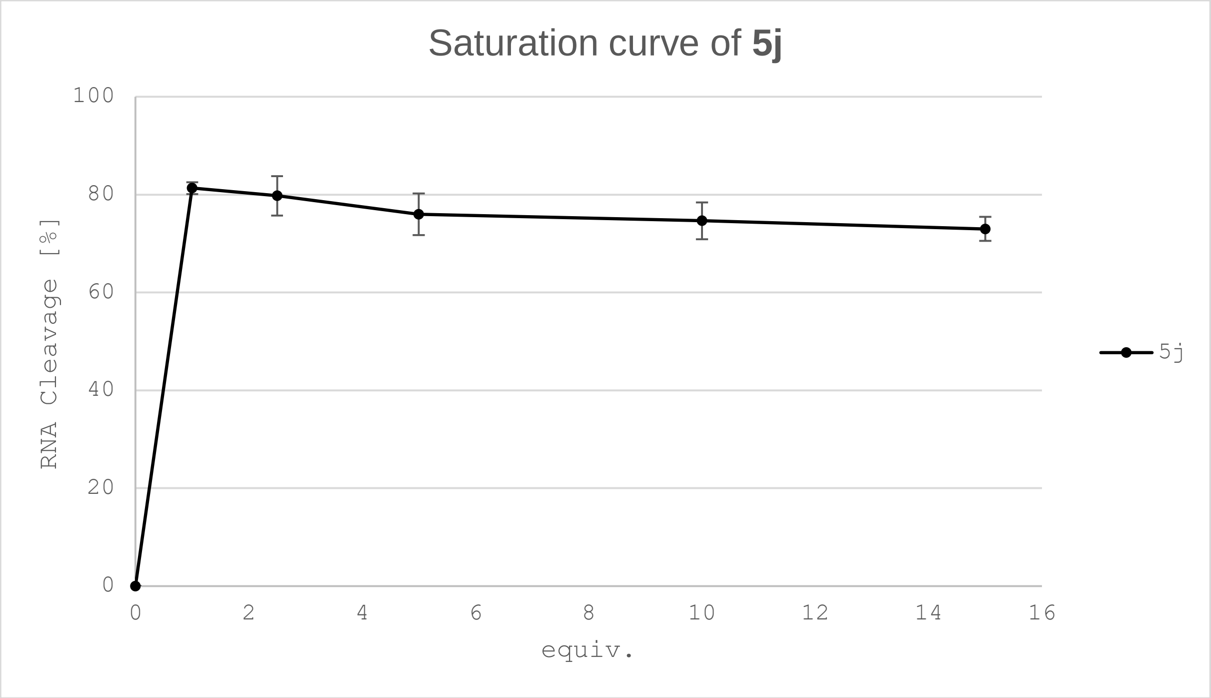

Figure S20: Saturation curve of conjugate **5j**. Conjugate **5j** (150 nM – 2.25 µM), RNA **8** (150 nM), 50 mM Tris-HCl pH 8.0, 100 mM NaCl, 37 °C, 20h.

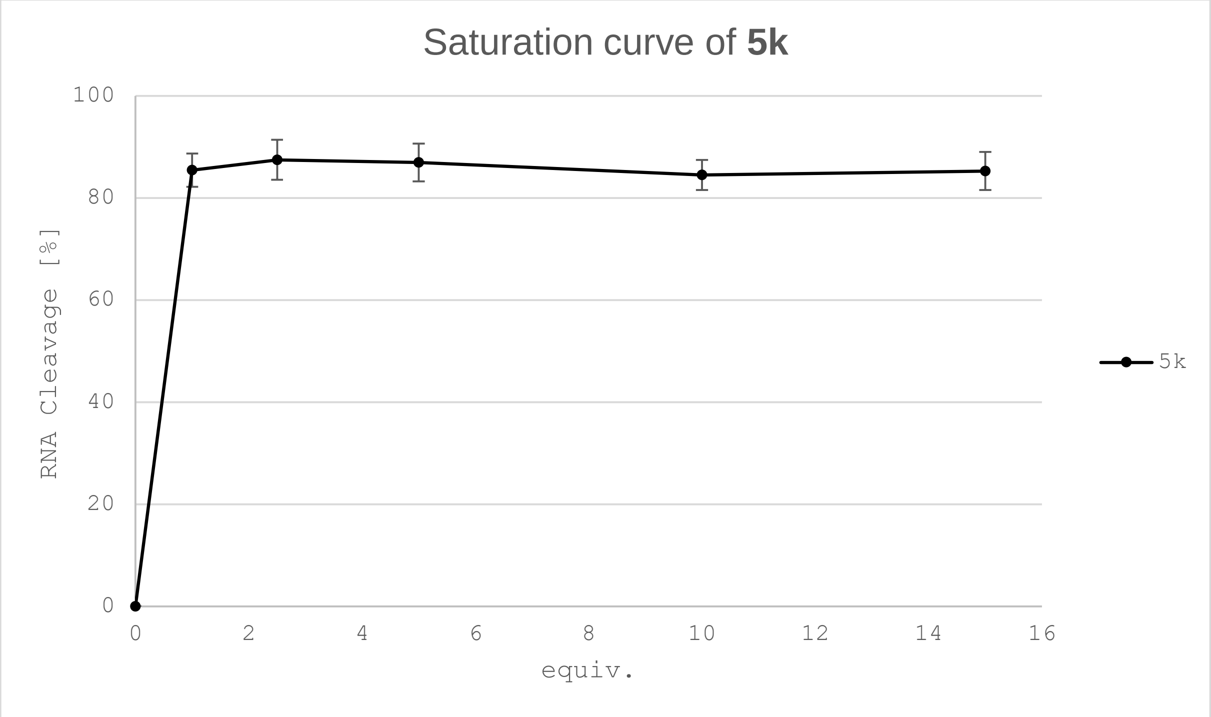

Figure S21: Saturation curve of conjugate **5k**. Conjugate **5k** (150 nM – 2.25 µM), RNA **8** (150 nM), 50 mM Tris-HCl pH 8.0, 100 mM NaCl, 37 °C, 20h.

Determination of k_{obs}

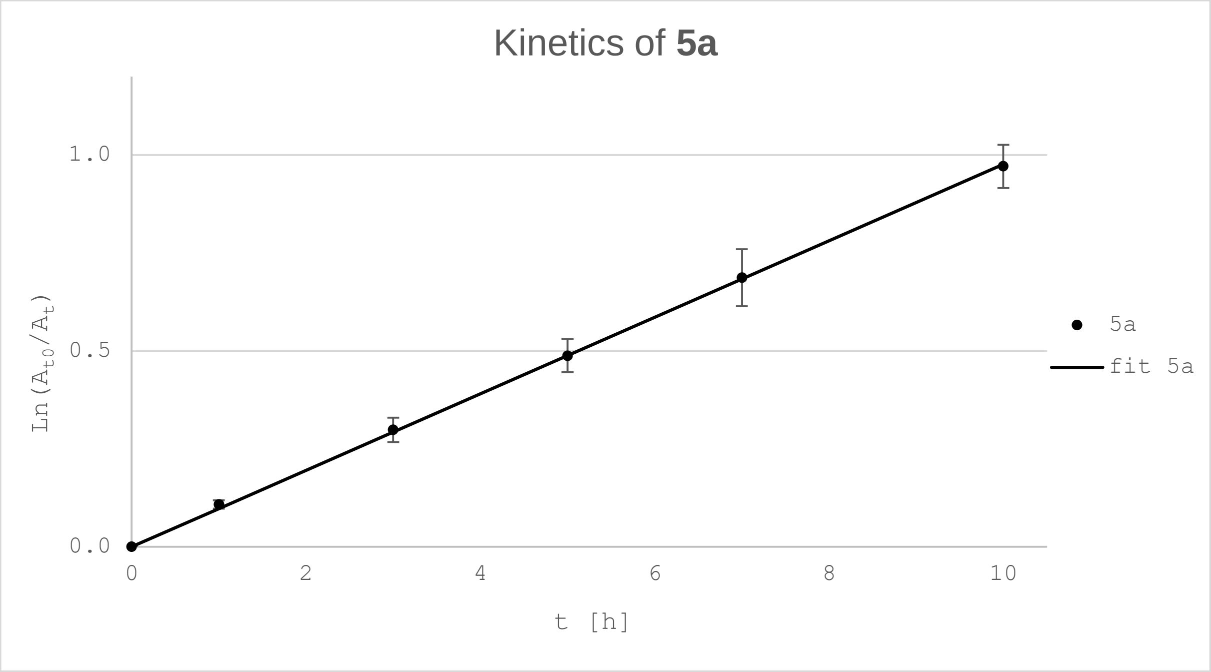

Figure S22: Kinetic measurements of conjugate **5a**. Conjugate **5a** (1.5 µM), RNA **6** (150 nM), 50 mM Tris-HCl pH 8.0, 100 mM NaCl, 37 °C.

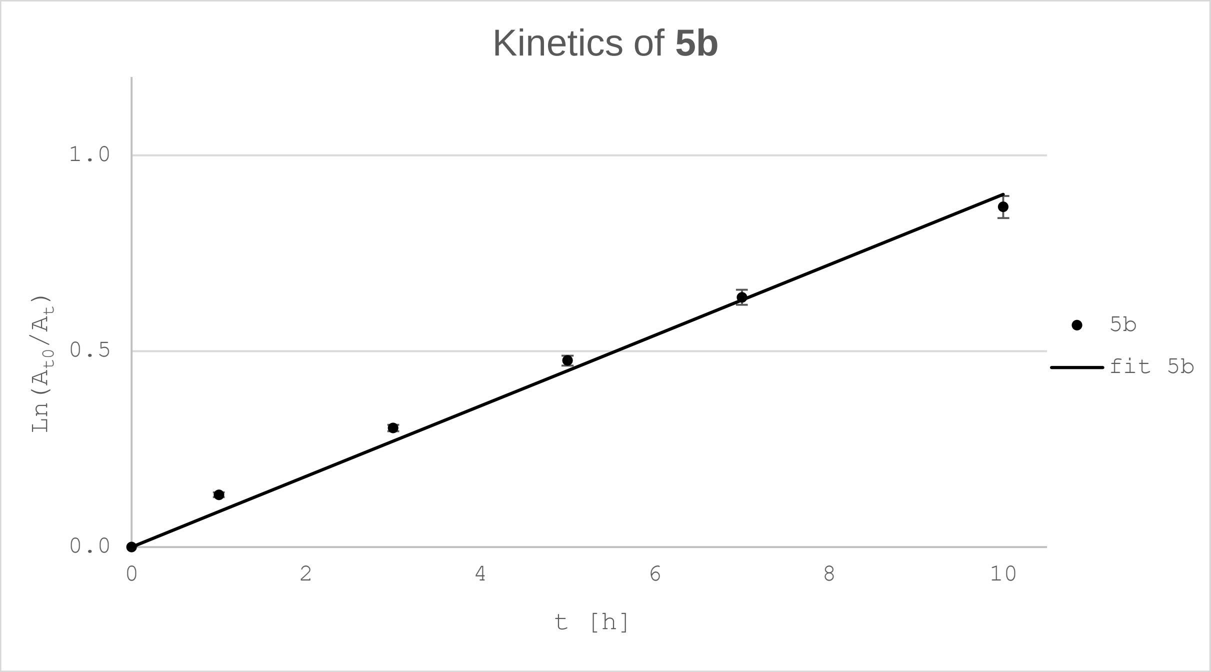

Figure S23: Kinetic measurements of conjugate **5b**. Conjugate **5b** (1.5 µM), RNA **6** (150 nM), 50 mM Tris-HCl pH 8.0, 100 mM NaCl, 37 °C.

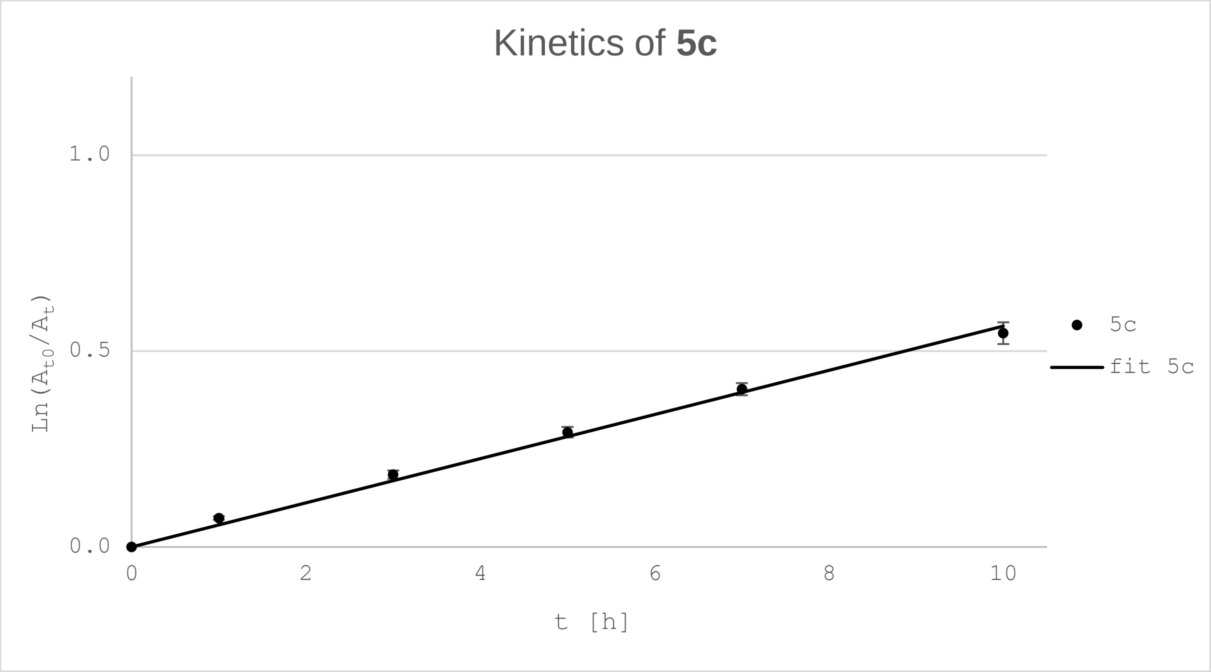

Figure S24: Kinetic measurements of conjugate **5c**. Conjugate **5c** (1.5 µM), RNA **6** (150 nM), 50 mM Tris-HCl pH 8.0, 100 mM NaCl, 37 °C.

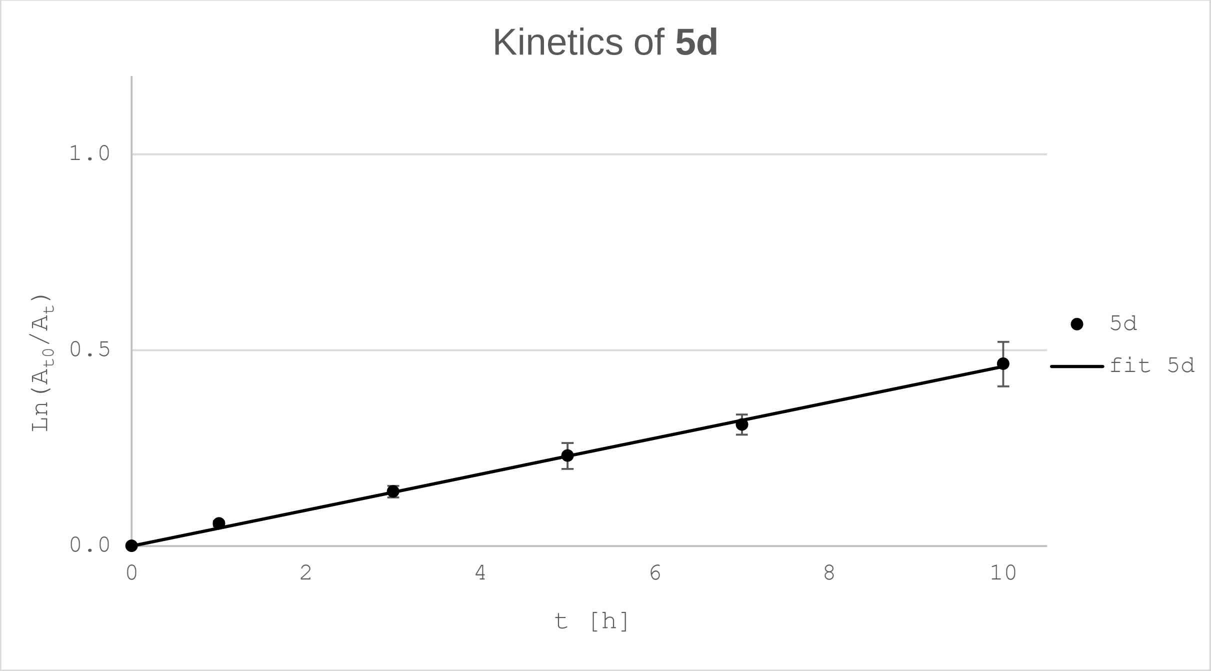

Figure S25: Kinetic measurements of conjugate **5d**. Conjugate **5d** (1.5 µM), RNA **6** (150 nM), 50 mM Tris-HCl pH 8.0, 100 mM NaCl, 37 °C.

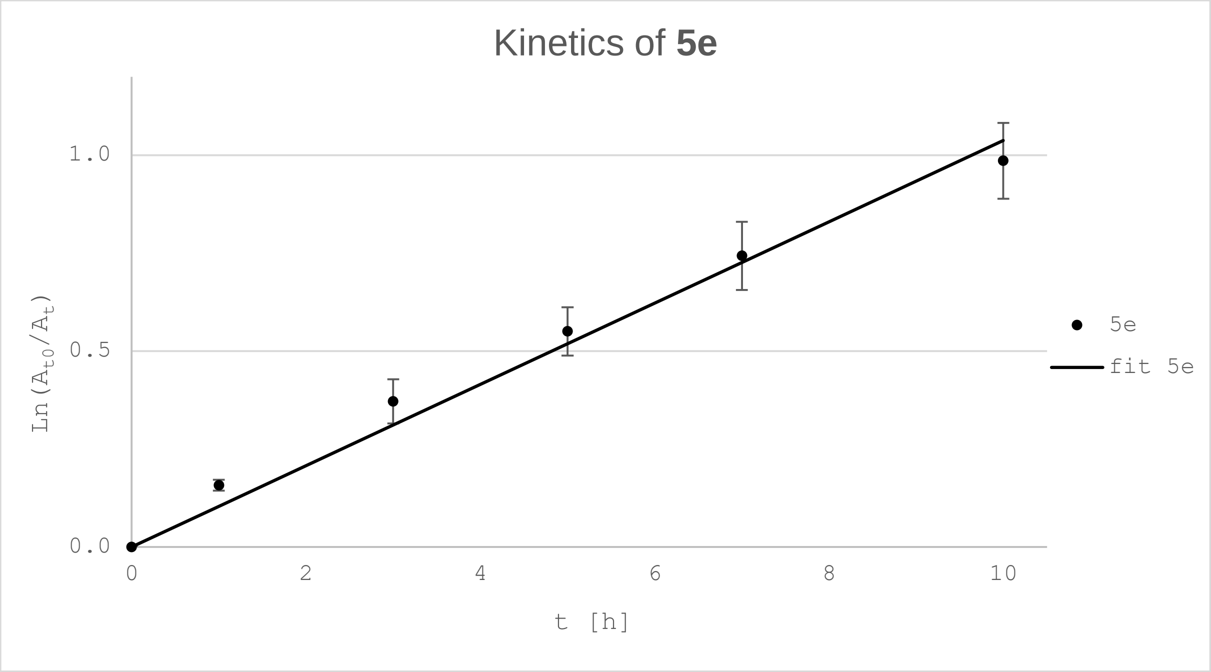

Figure S26: Kinetic measurements of conjugate **5e**. Conjugate **5e** (1.5 µM), RNA **6** (150 nM), 50 mM Tris-HCl pH 8.0, 100 mM NaCl, 37 °C.

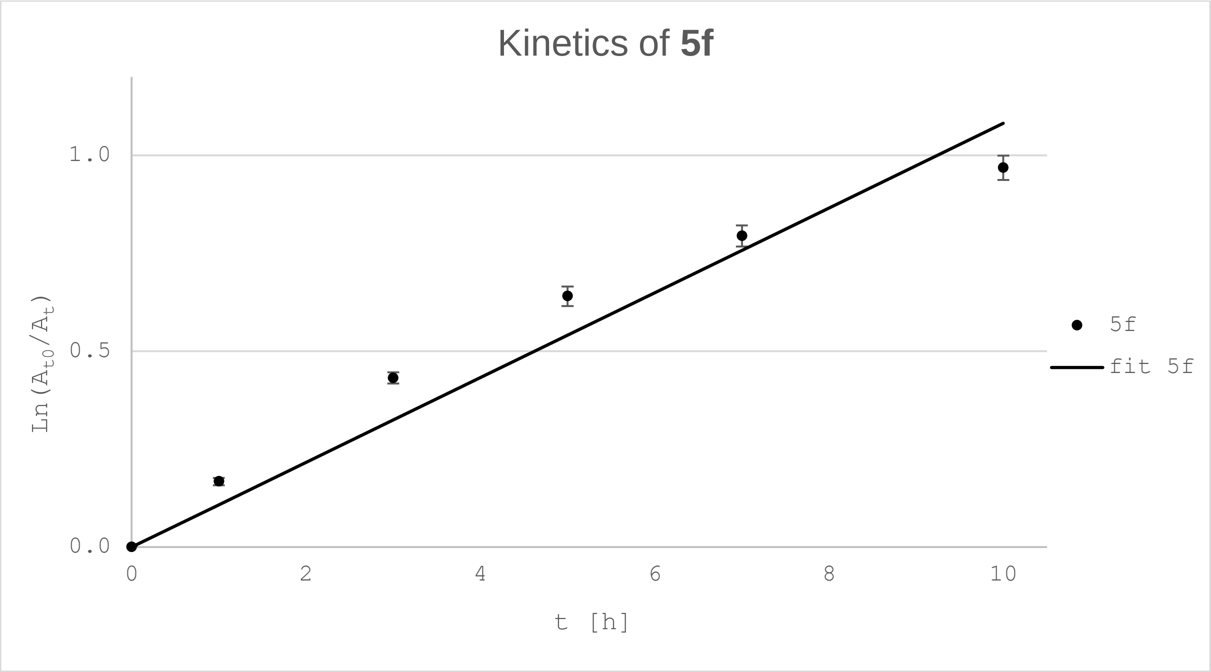

Figure S27: Kinetic measurements of conjugate **5f**. Conjugate **5f** (1.5 µM), RNA **6** (150 nM), 50 mM Tris-HCl pH 8.0, 100 mM NaCl, 37 °C.

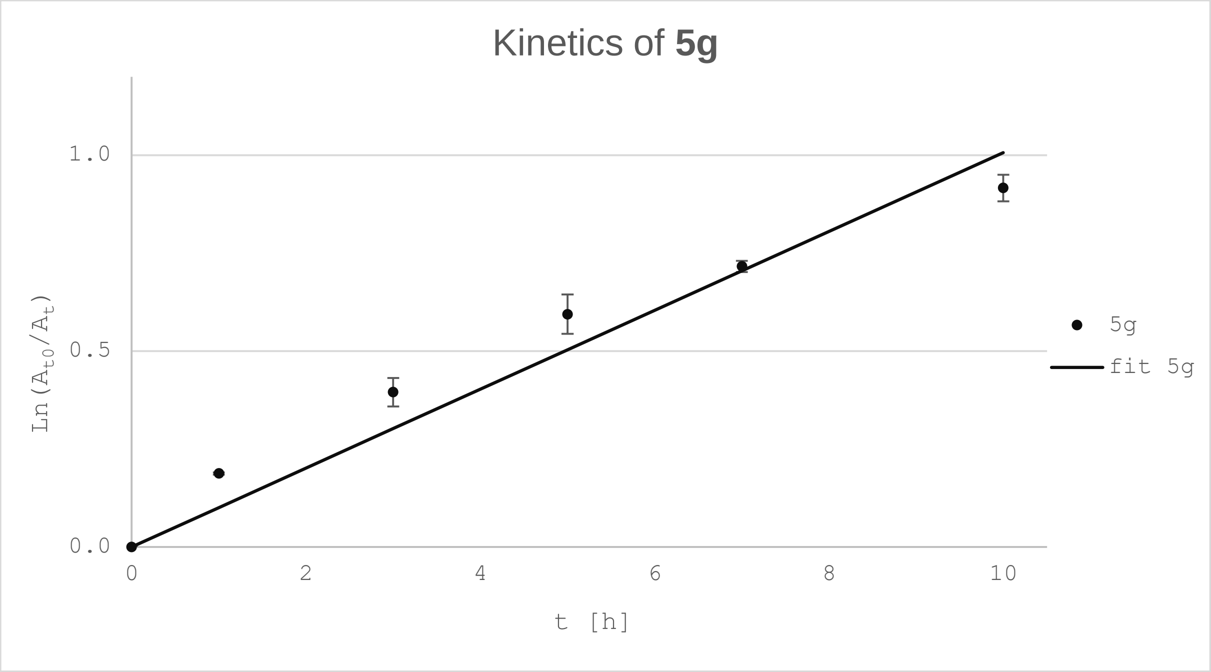

Figure S28: Kinetic measurements of conjugate **5g**. Conjugate **5g** (1.5 µM), RNA **6** (150 nM), 50 mM Tris-HCl pH 8.0, 100 mM NaCl, 37 °C.

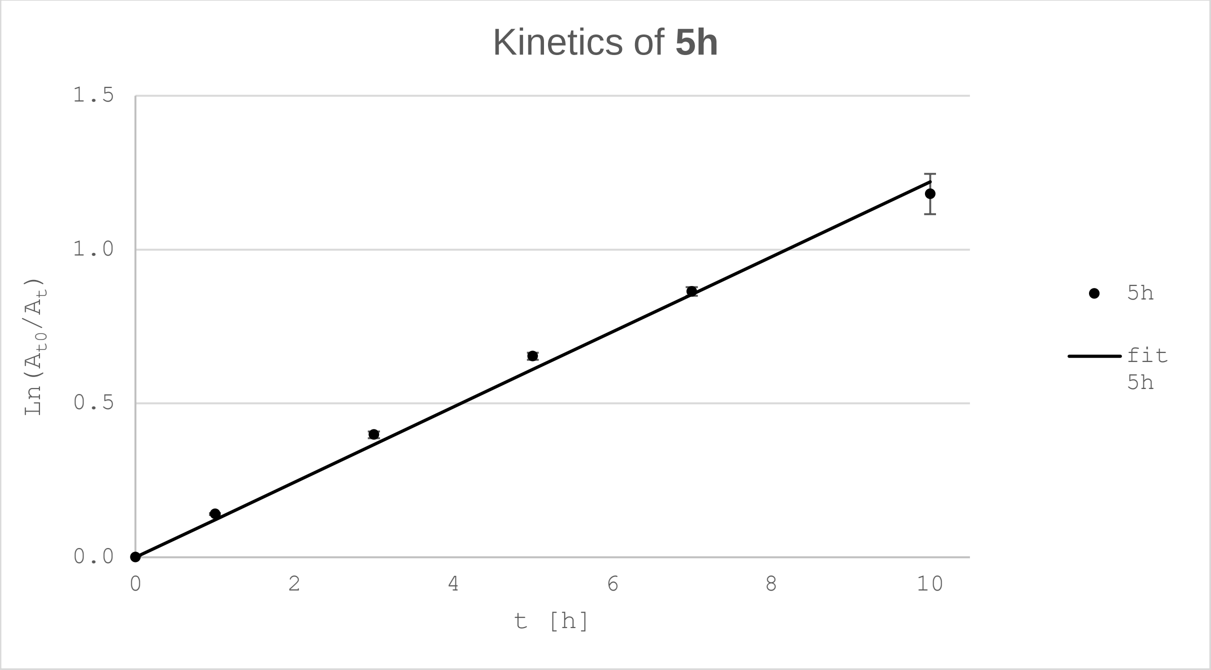

Figure S29: Kinetic measurements of conjugate **5h**. Conjugate **5h** (1.5 µM), RNA **7** (150 nM), 50 mM Tris-HCl pH 8.0, 100 mM NaCl, 37 °C.

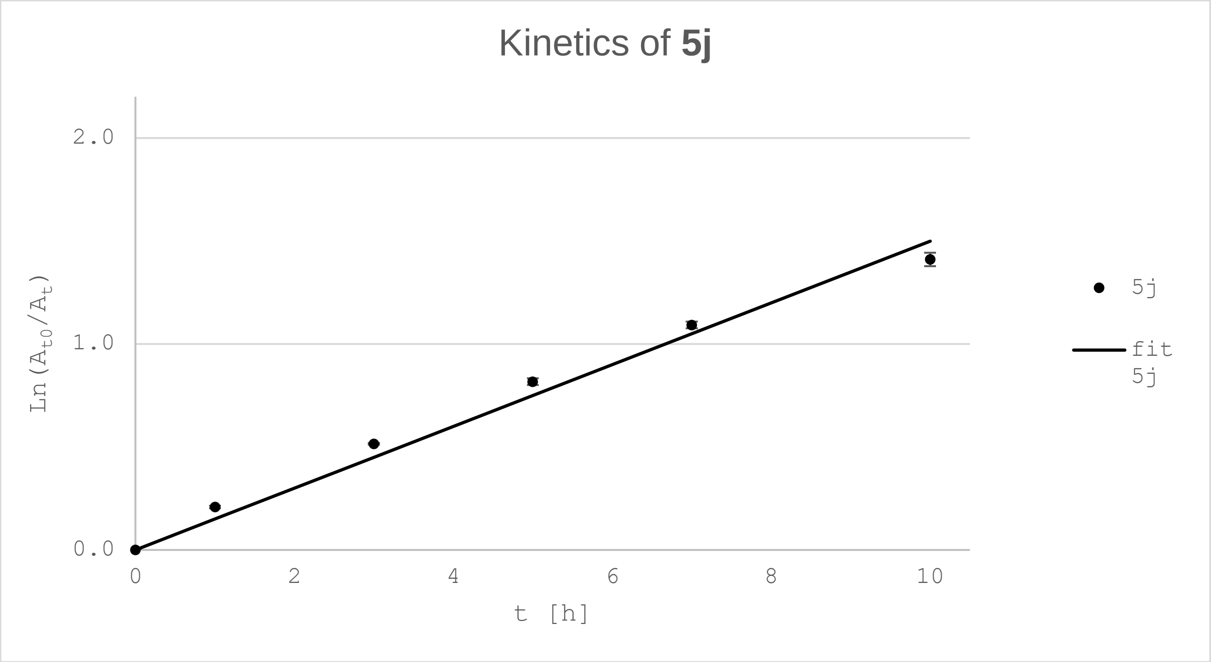

Figure S30: Kinetic measurements of conjugate **5j**. Conjugate **5j** (1.5 µM), RNA **8** (150 nM), 50 mM Tris-HCl pH 8.0, 100 mM NaCl, 37 °C.

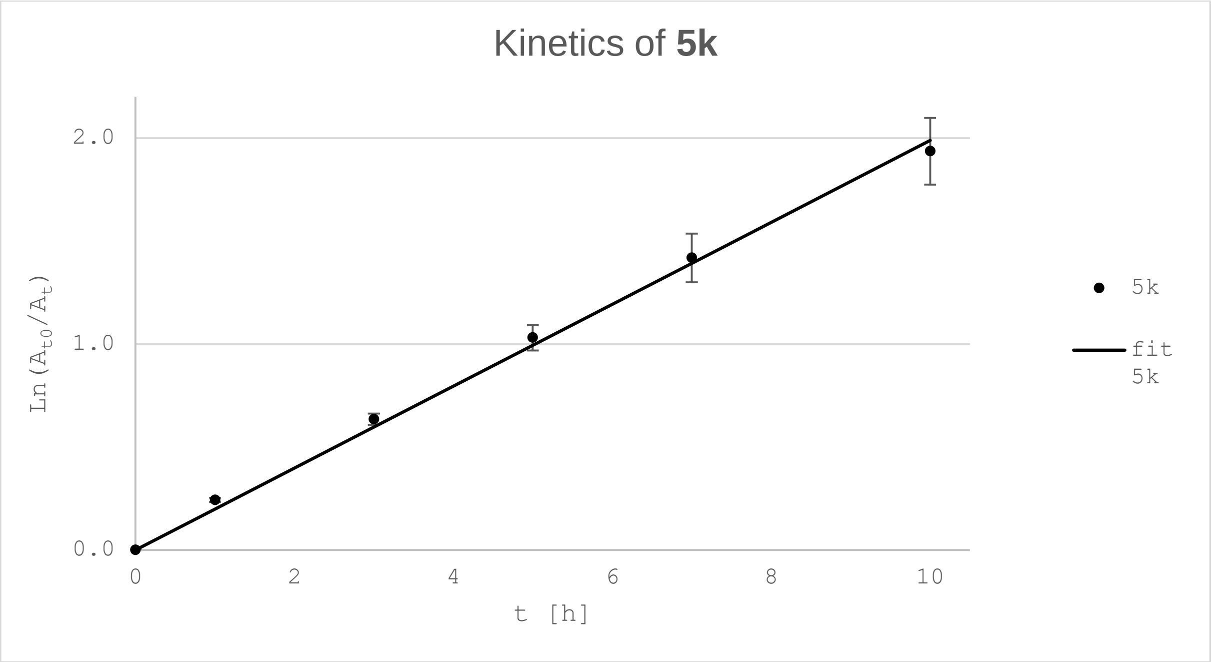

Figure S31: Kinetic measurements of conjugate **5k**. Conjugate **5k** (1.5 µM), RNA **8** (150 nM), 50 mM Tris-HCl pH 8.0, 100 mM NaCl, 37 °C.

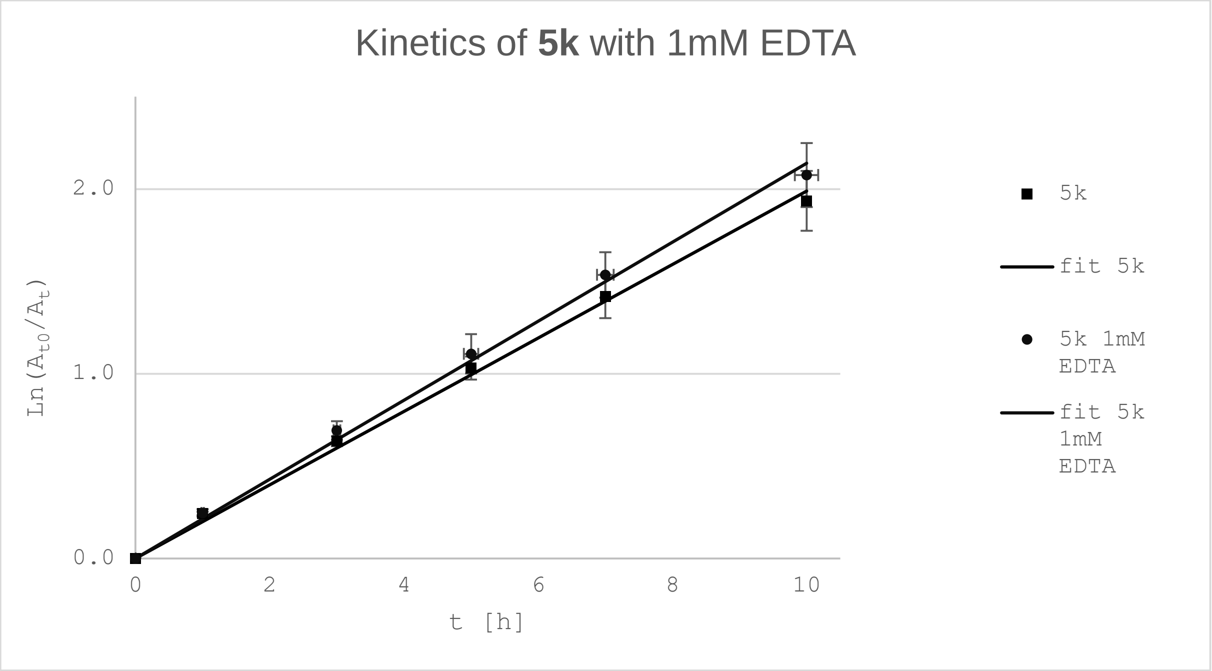

Figure S32: Kinetic measurements of conjugate **5k** without (black squares) and with 1 mM EDTA (black circles). Conjugate **5k** (1.5 µM), RNA **8** (150 nM), 50 mM Tris-HCl pH 8.0, 100 mM NaCl, 37 °C.

Substrate specificity of conjugates

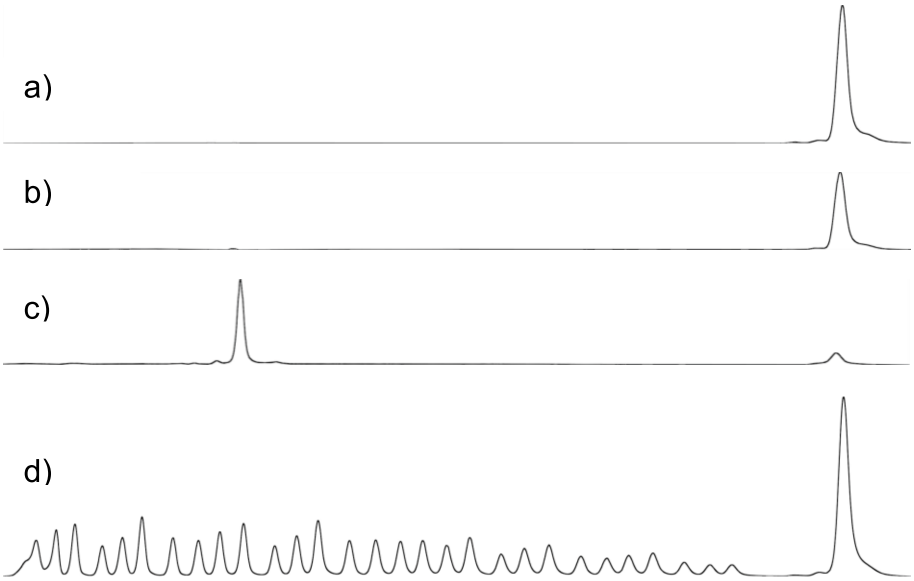

Figure S33: Conjugate **5g** but not **5h** and **5k** cleaves RNA **6**. a) **5h** and RNA **6**, b) **5k** and RNA **6**, c) **5g** and RNA **6**, d) hydrolysis ladder of RNA **6**. RNA **6** (150 nM), conjugates **5** (750 nM), 20 h at 37 °C (50 mM Tris buffer pH 8, 100 mM NaCl).

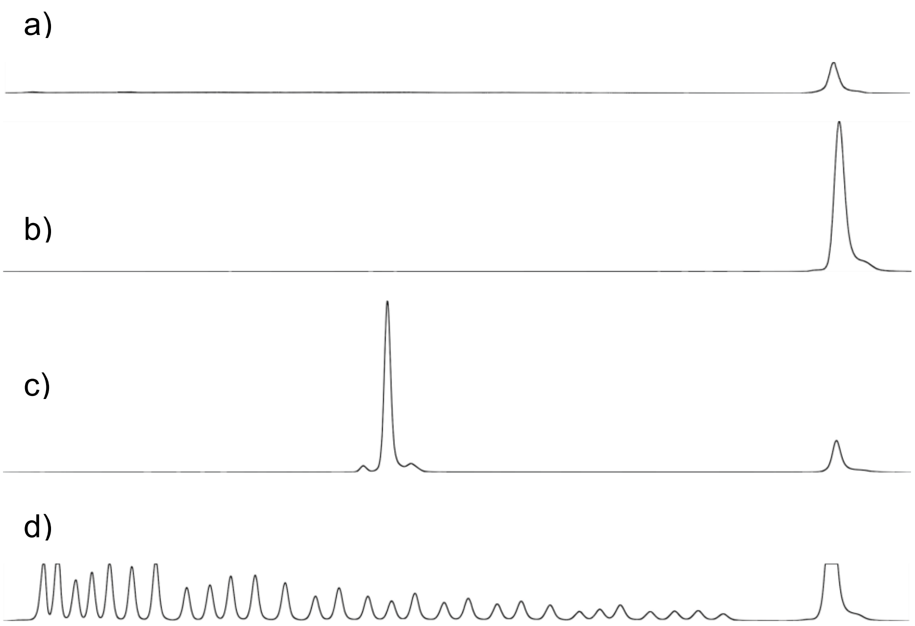

Figure S34: Conjugate **5h** but not **5g** and **5k** cleaves RNA **7**. a) **5g** and RNA **7**, b) **5k** and RNA **7**, c) **5h** and RNA **7**, d) hydrolysis ladder of RNA **7**. RNA **7** (150 nM), conjugates **5** (750 nM), 20 h at 37 °C (50 mM Tris buffer pH 8, 100 mM NaCl).

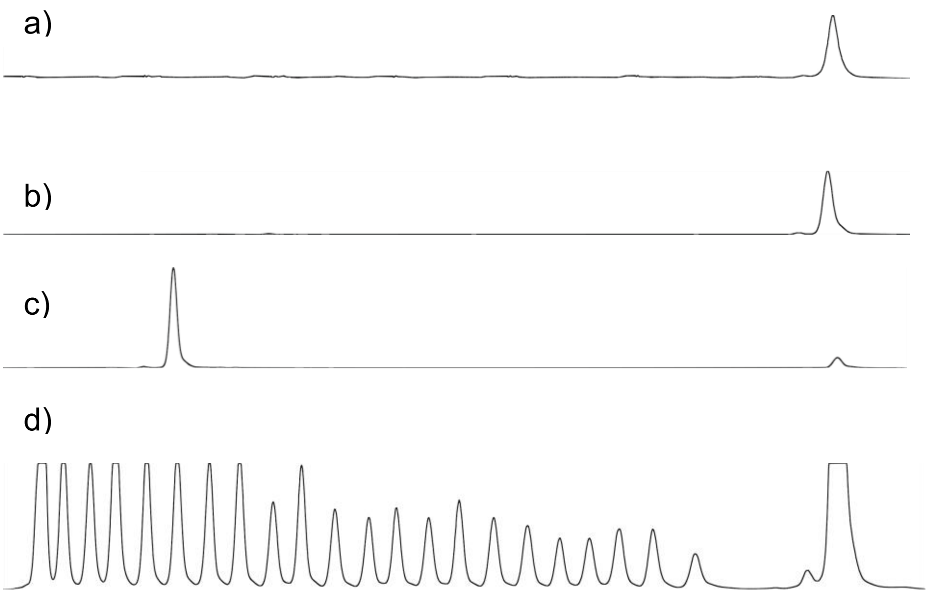

Figure S35: Conjugate **5k** but not **5g** and **5h** cleaves RNA **8**. a) **5g** and RNA **8**, b) **5h** and RNA **8**, c) **5k** and RNA **8**, d) hydrolysis ladder of RNA **8**. RNA **8** (150 nM), conjugates **5** (750 nM), 20 h at 37 °C (50 mM Tris buffer pH 8, 100 mM NaCl).

Substrate<br>Conjugate	RNA **6**	RNA **7**	RNA **8**
**5g**			
**5h**			
**5k**			

Temperature dependence of RNA cleavage by conjugate 5a

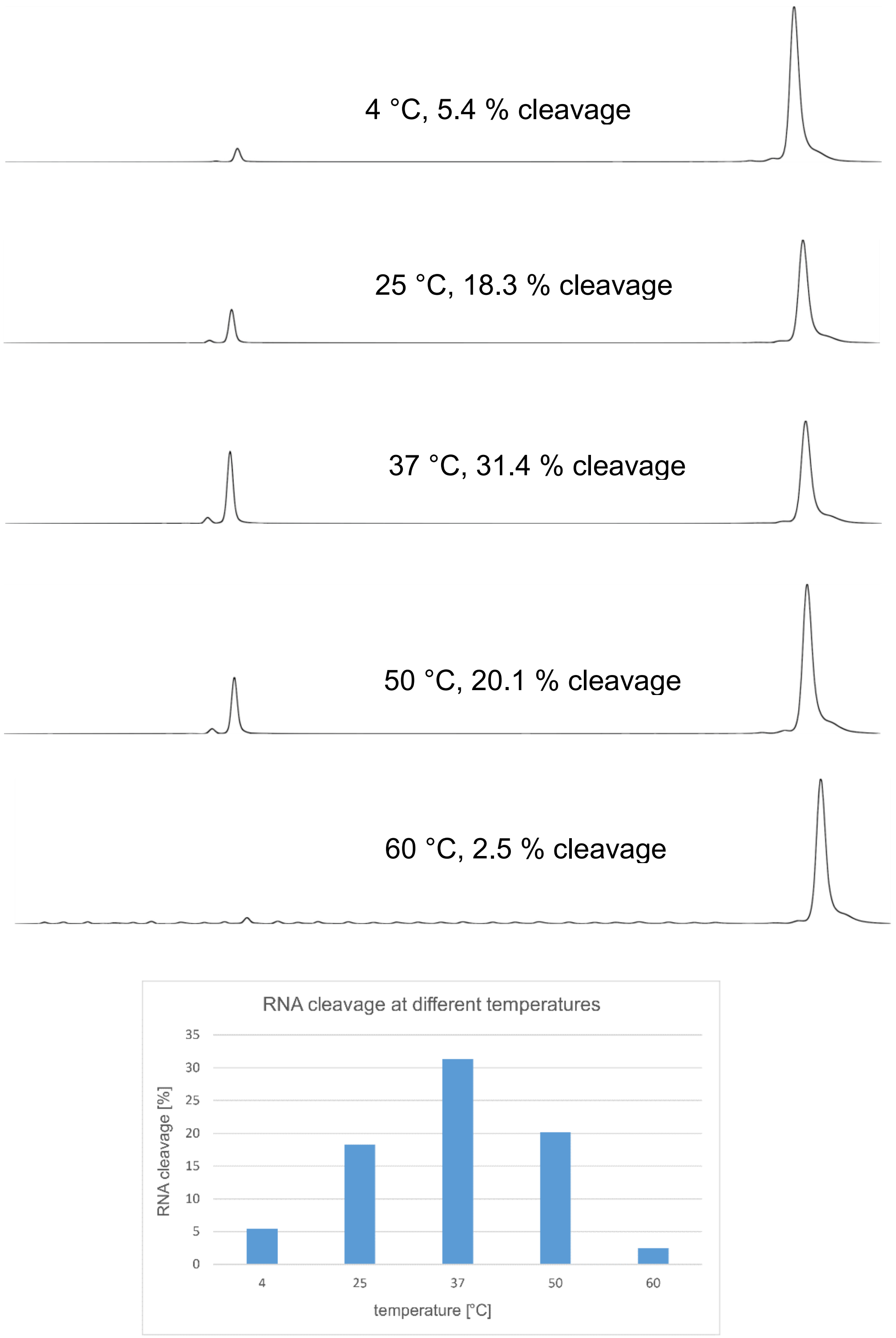

Figure S36: Temperature dependence of RNA cleavage by conjugate **5a**. 4 h of incubation at different temperatures, Tris buffer pH 8.0, 100 mM NaCl; 150 nM **6**, 10 equiv. of **5a**. Cleavage yields are mean values from three independent experiments.

HPLC chromatograms and mass spectra

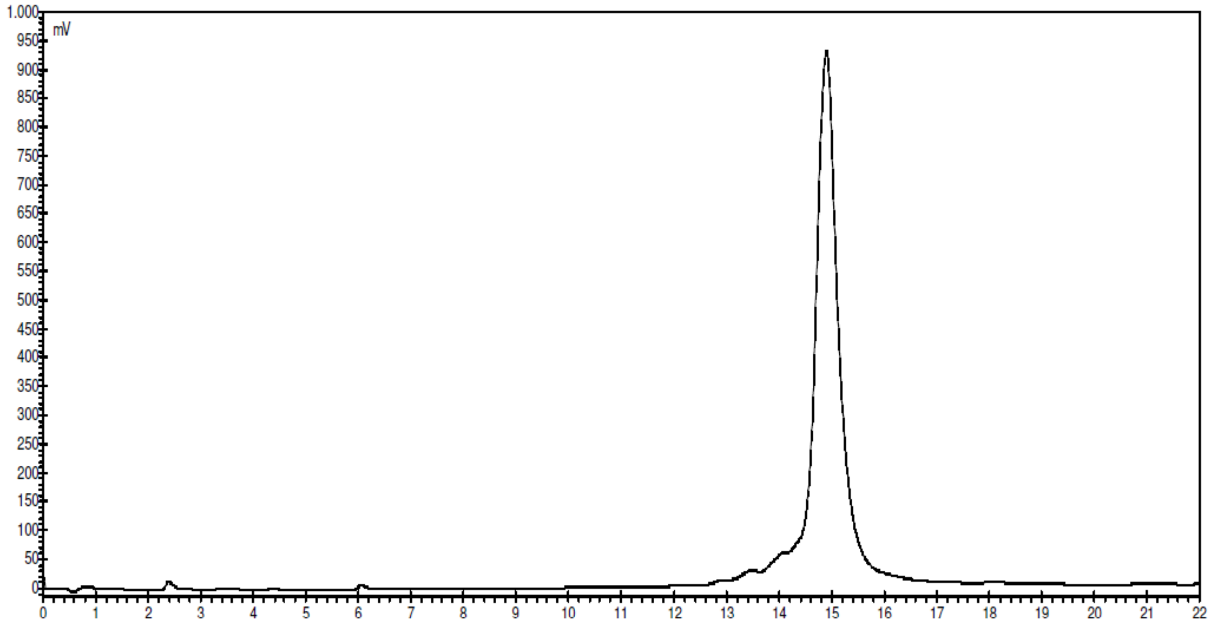

Figure S37: HPLC chromatogram of conjugate **5a**.

Conjugate **5a** calculated exact mass: 4921.12; found: 4920.97

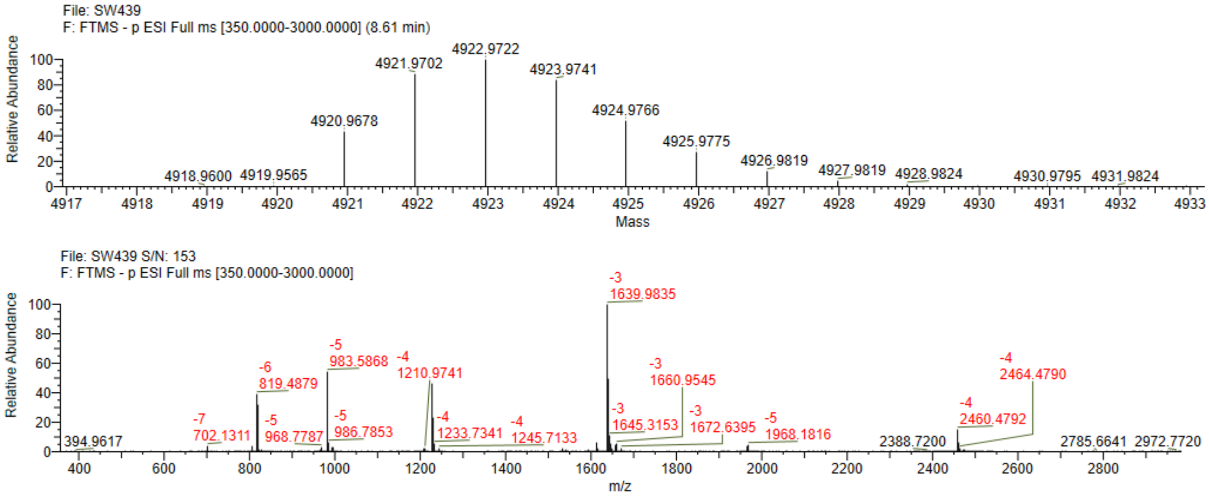

Figure S38: LC-MS spectra of conjugate **5a**.

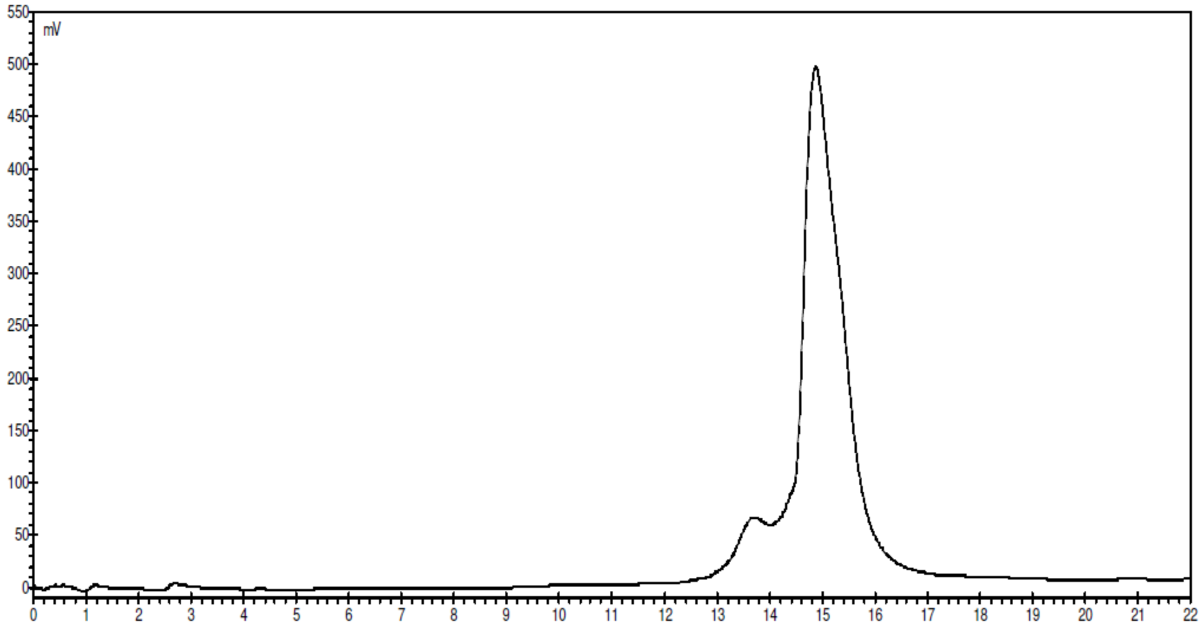

Figure S39: HPLC chromatogram of conjugate **5b**.

Conjugate **5b** calculated exact mass: 4977.11; found: 4976.9

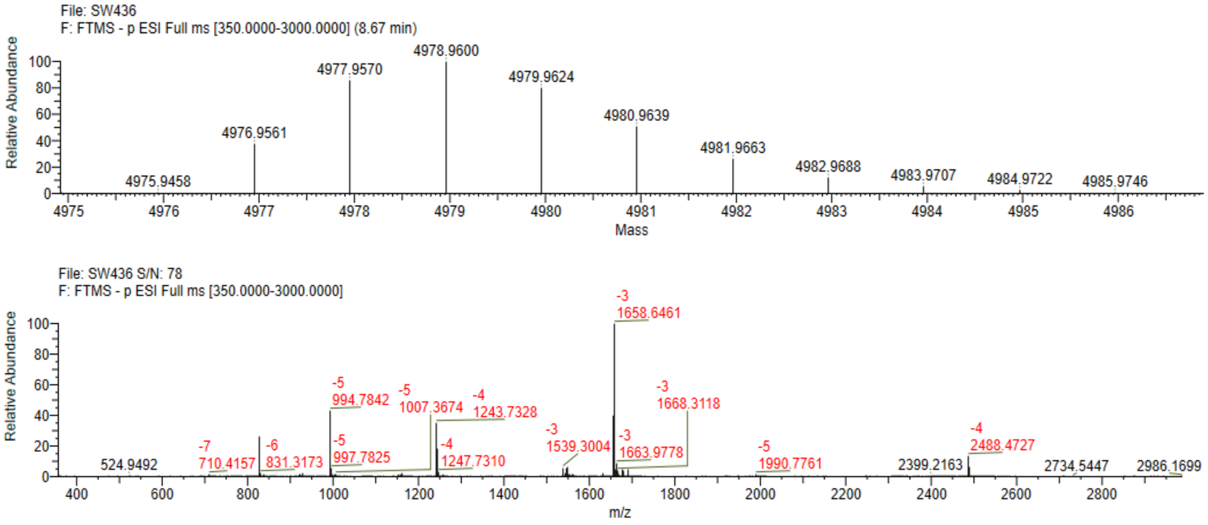

Figure S40: LC-MS spectra of conjugate **5b**.

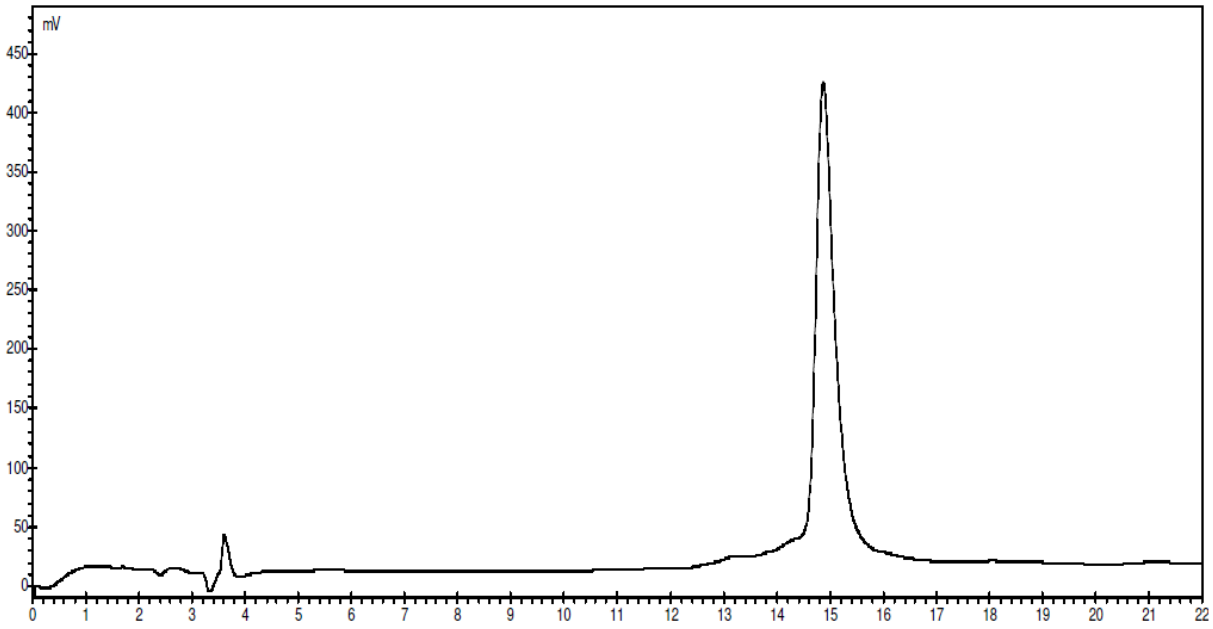

Figure S41: HPLC chromatogram of conjugate **5c**.

*Conjugate **5c** calculated exact mass: 4949.11; found: 4948.97*

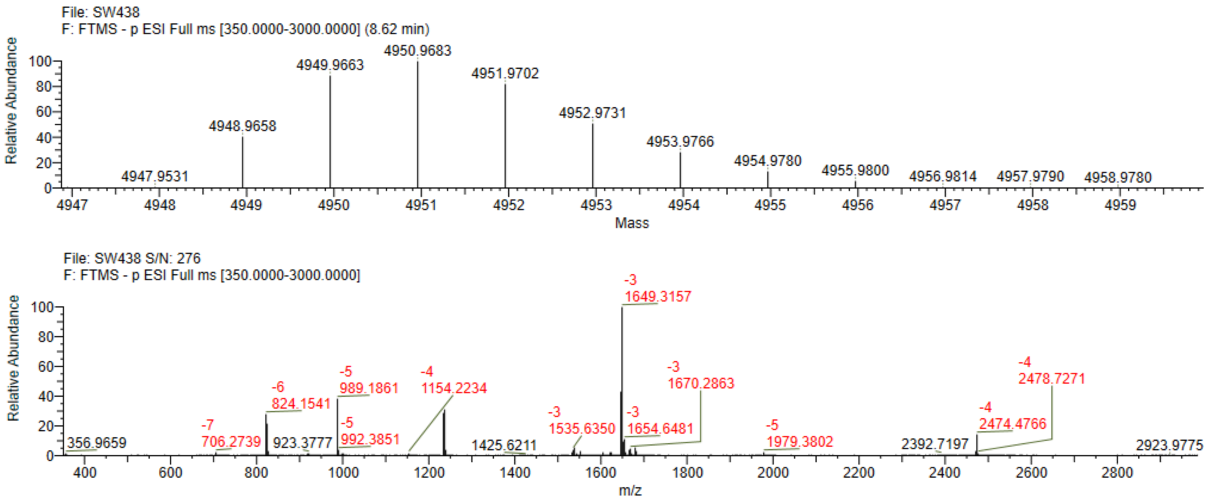

Figure S42: LC-MS spectra of conjugate **5c**.

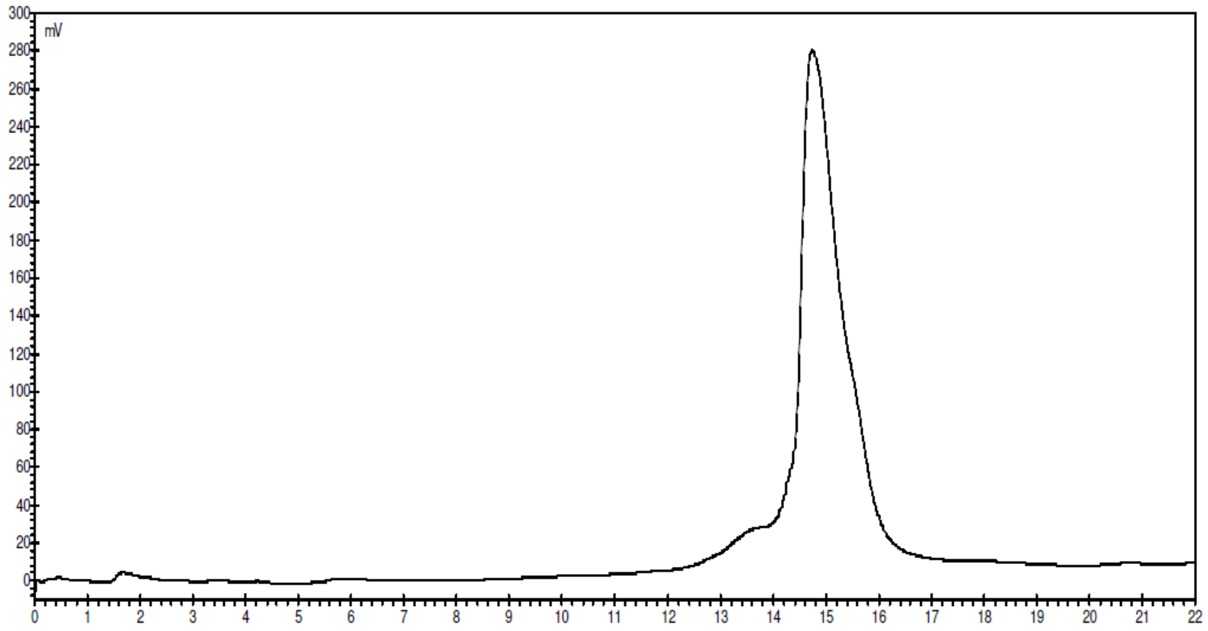

Figure S43: HPLC chromatogram of conjugate **5d**.

Conjugate **5d** calculated exact mass: 4949.11; found: 4948.97

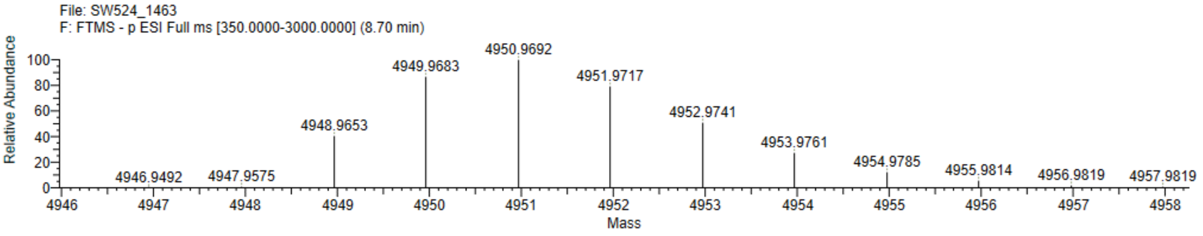

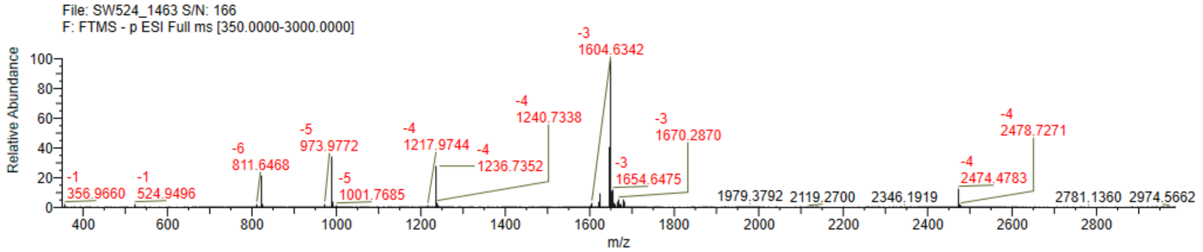

Figure S44: LC-MS spectra of conjugate **5d**.

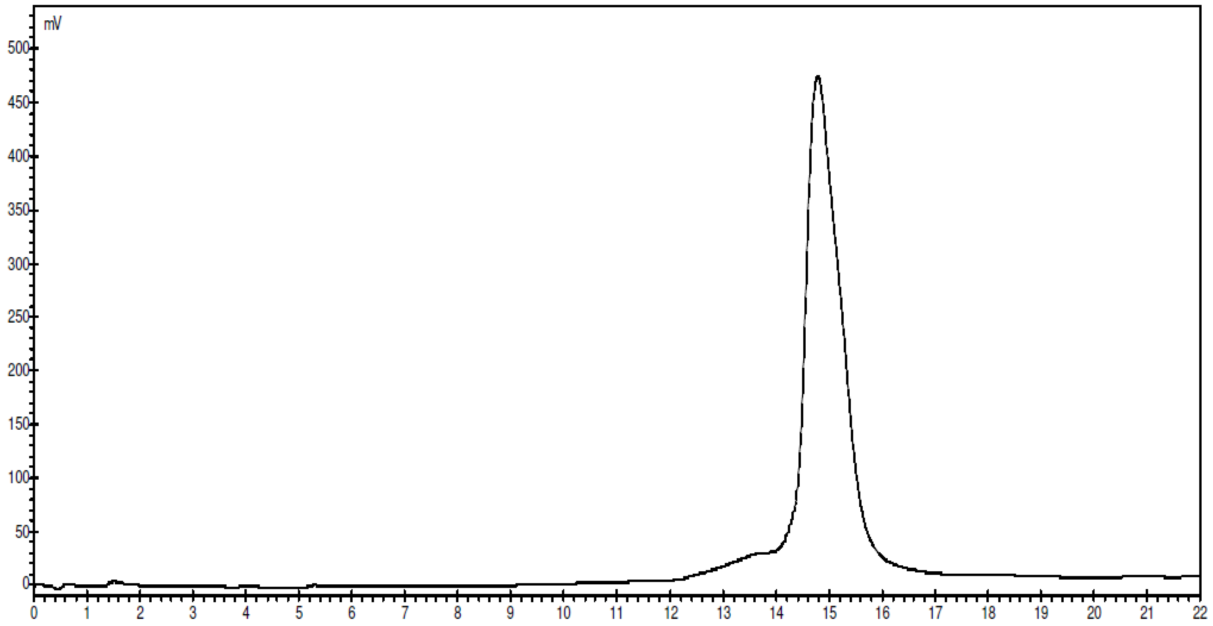

Figure S45: HPLC chromatogram of conjugate **5e**.

Conjugate **5e** calculated exact mass: 4949.11; found: 4948.97

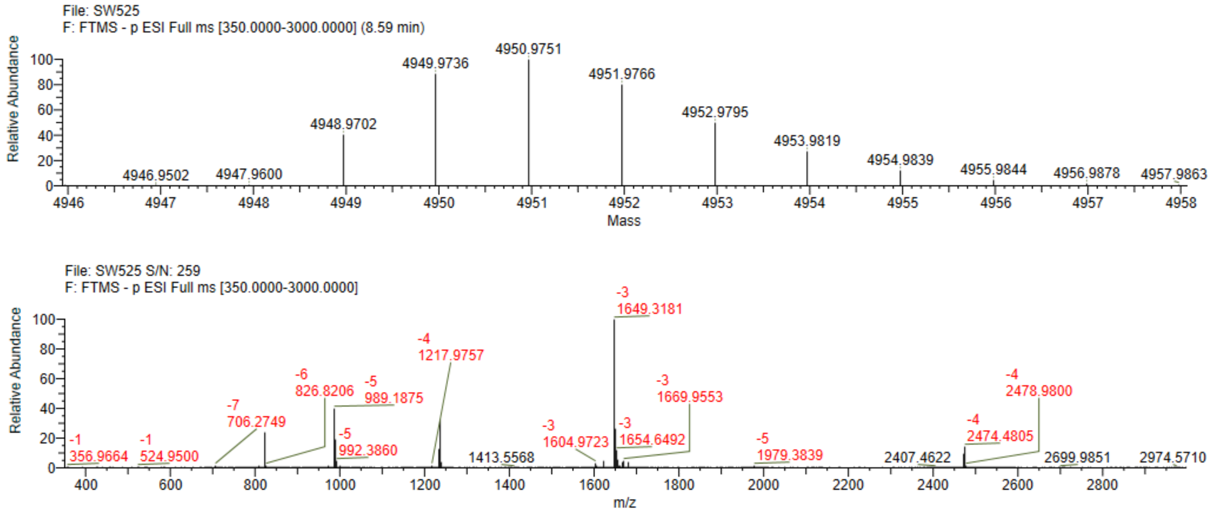

Figure S46: LC-MS spectra of conjugate **5e**.

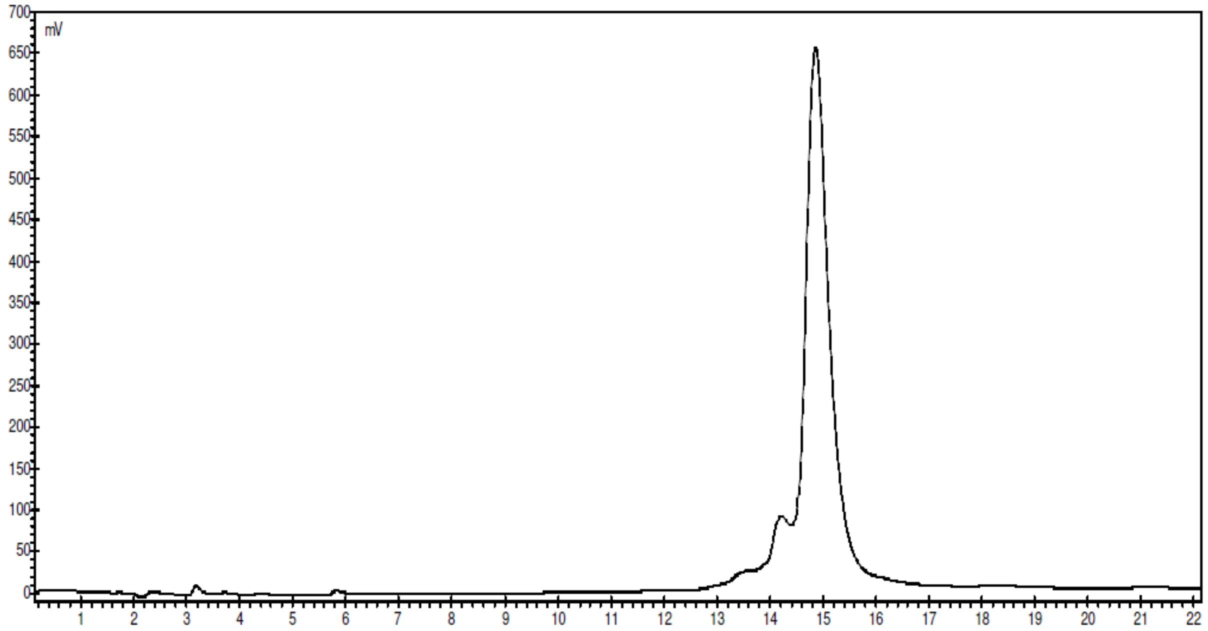

Figure S47: HPLC chromatogram of conjugate **5f**.

Conjugate **5f** calculated exact mass: 4977.11; found: 4976.96

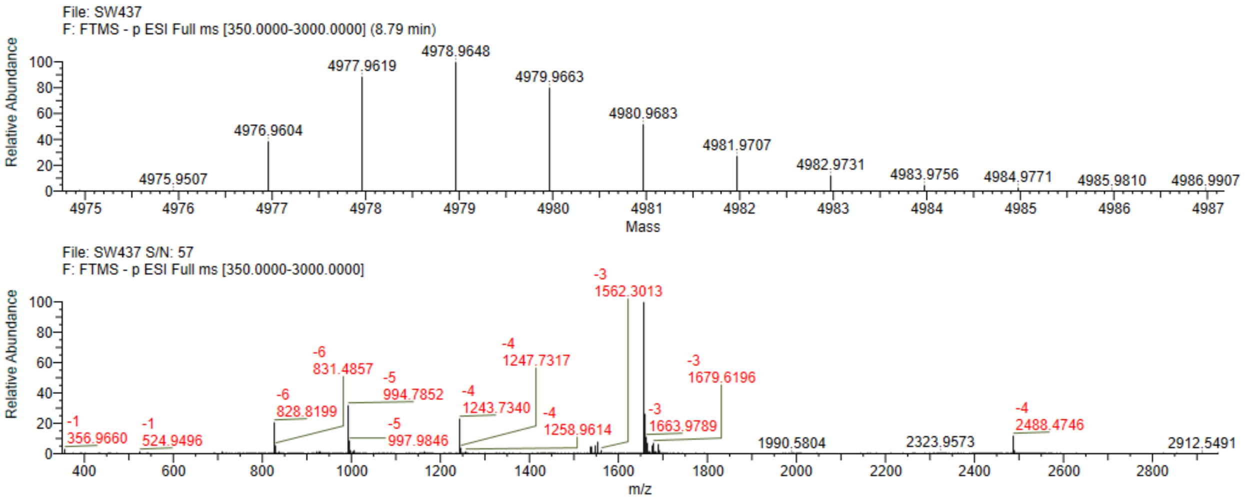

Figure S48: LC-MS spectra of conjugate **5f**.

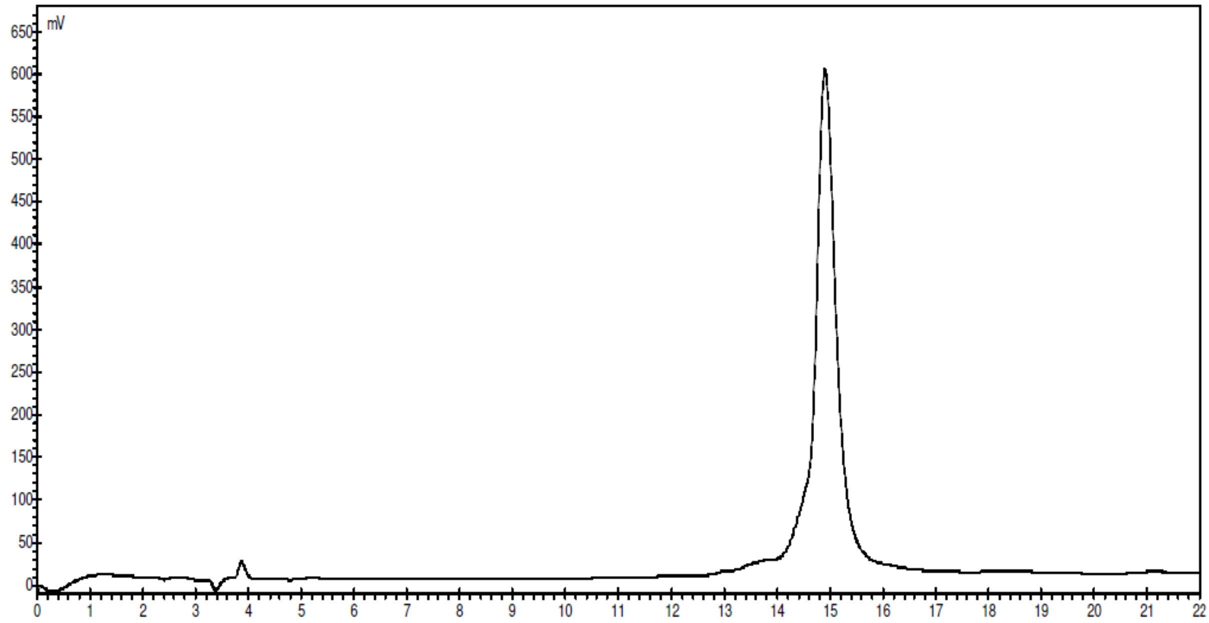

Figure S49: HPLC chromatogram of conjugate **5g**.

Conjugate **5g** calculated exact mass: 5061.09; found: 5060.94

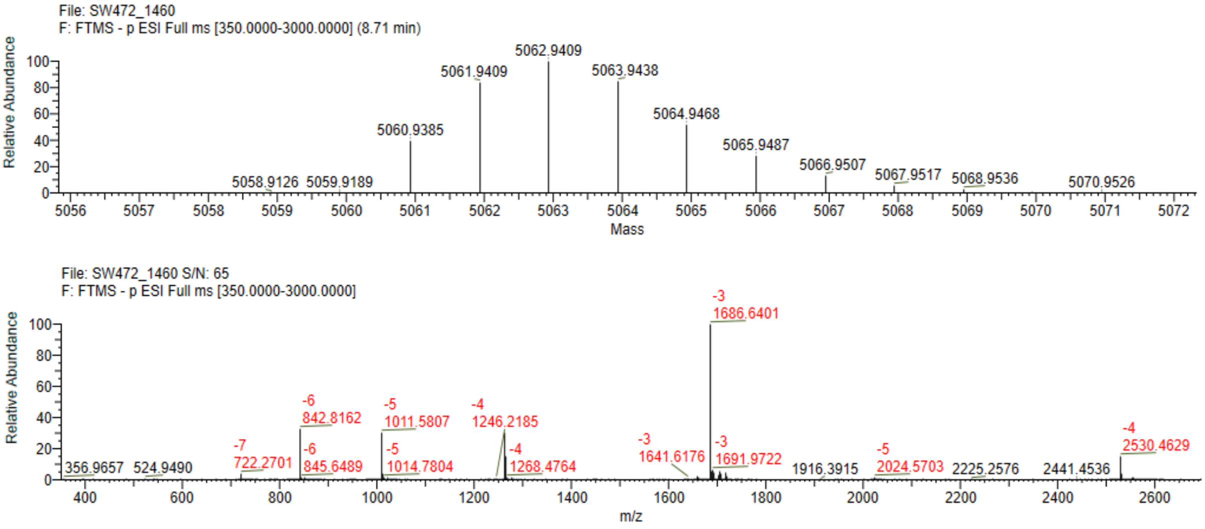

Figure S50: LC-MS spectra of conjugate **5g**.

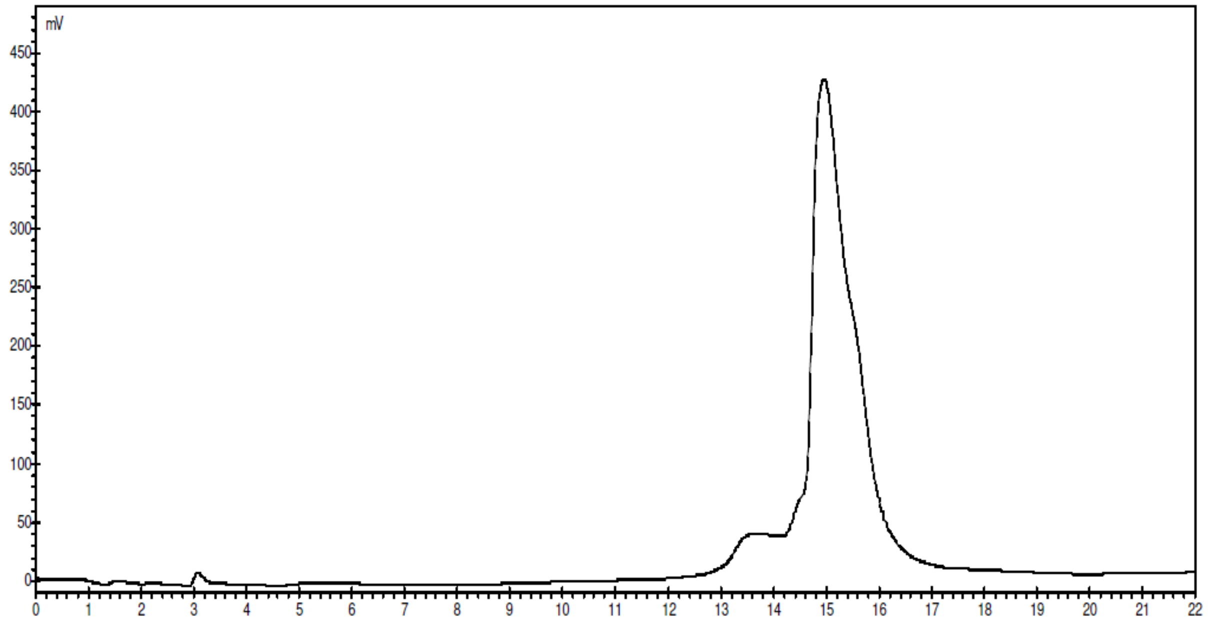

Figure S51: HPLC chromatogram of conjugate **5h**.

Conjugate **5h** calculated exact mass: 4718.06; found: 4717.92

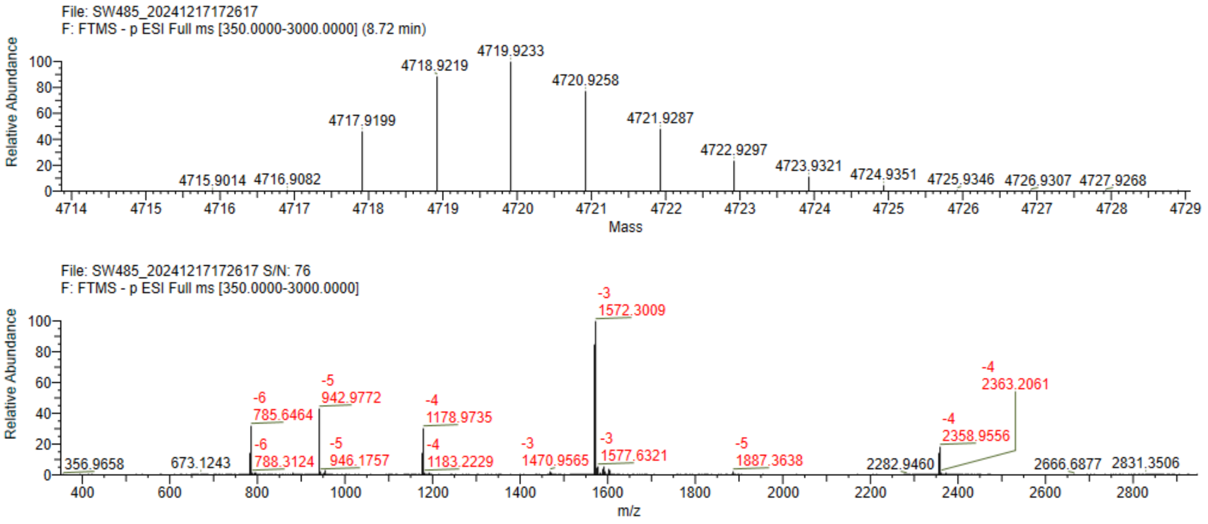

Figure S52: LC-MS spectra of conjugate **5h**.

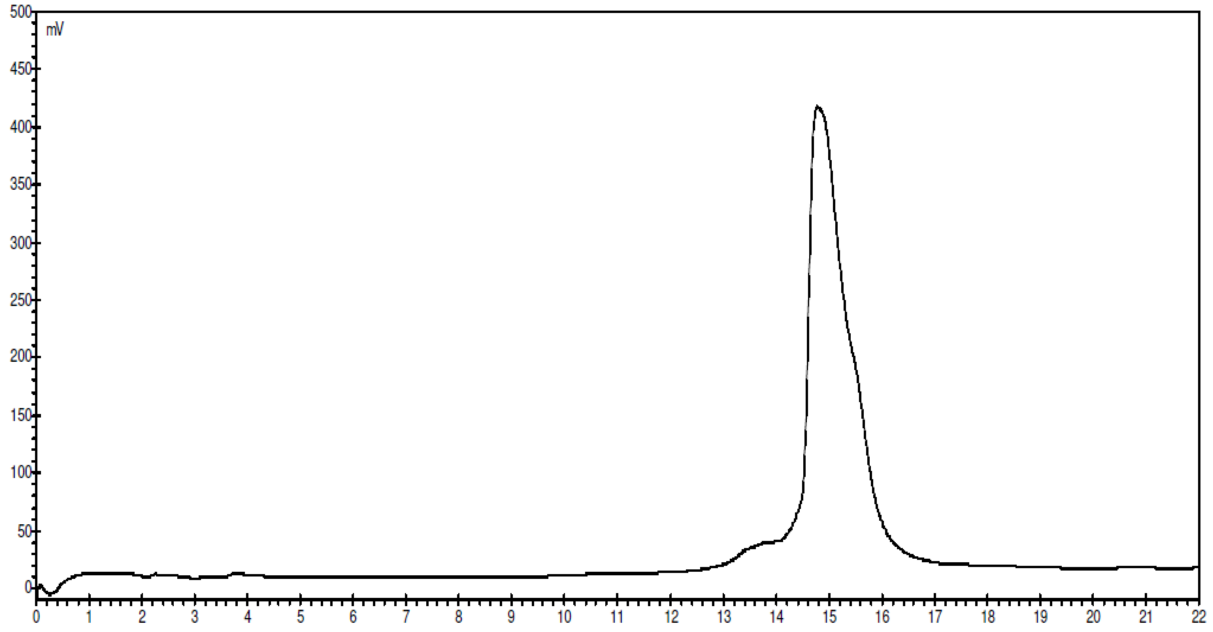

Figure S53: HPLC chromatogram of conjugate **5j**.

Conjugate **5j** calculated exact mass: 5366.15; found: 5365.98

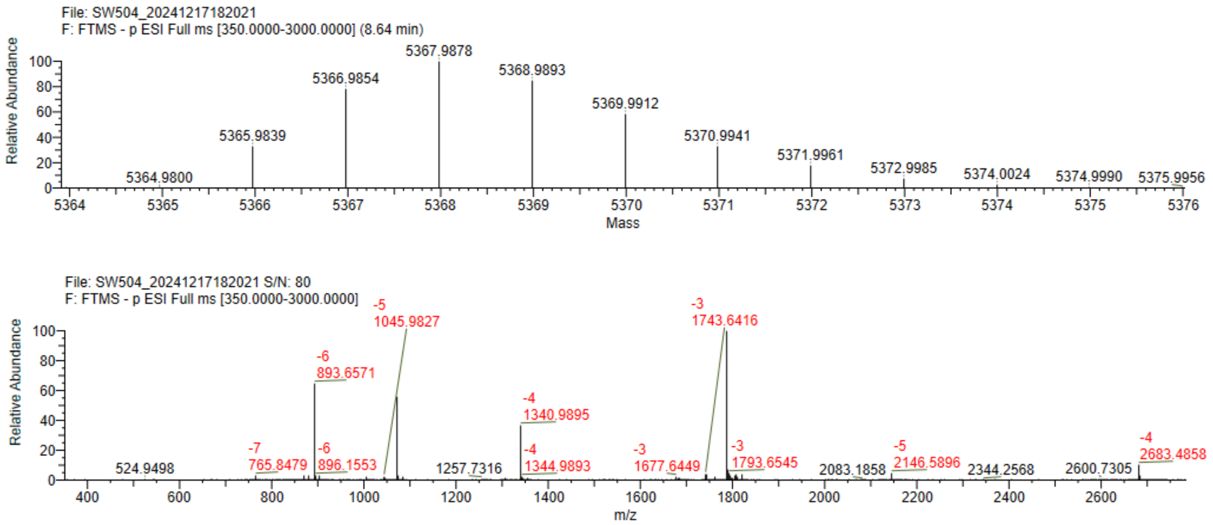

Figure S54: LC-MS spectra of conjugate **5j**.

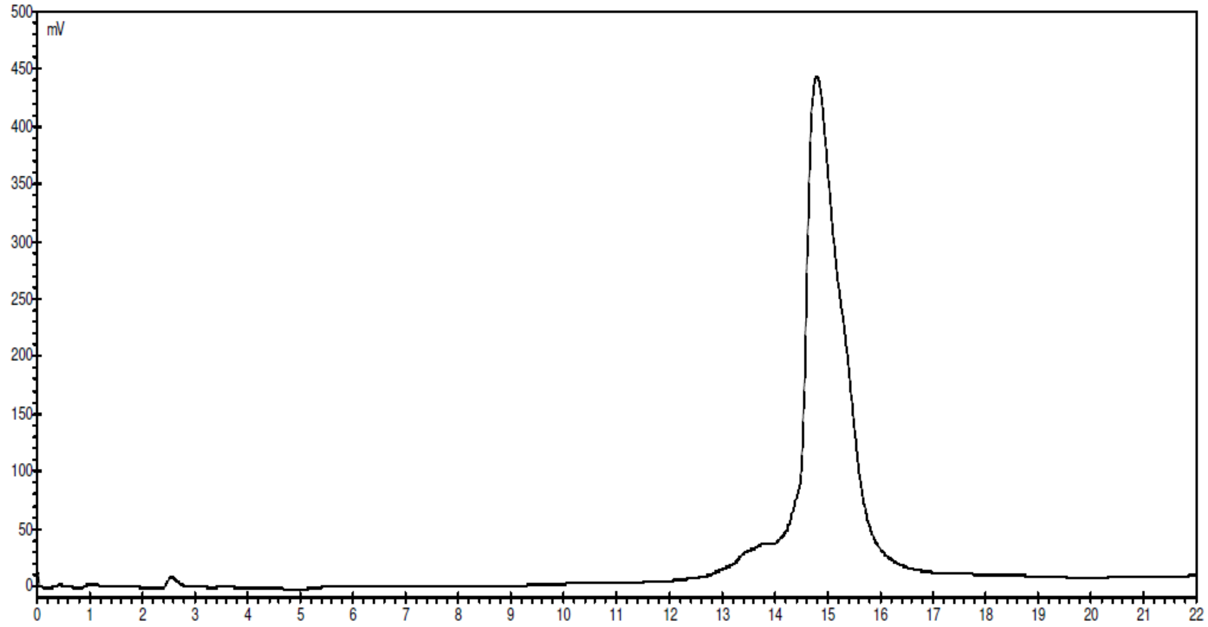

Figure S55: HPLC chromatogram of conjugate **5k**.

Conjugate **5k** calculated exact mass: 5366.15; found: 5365.9

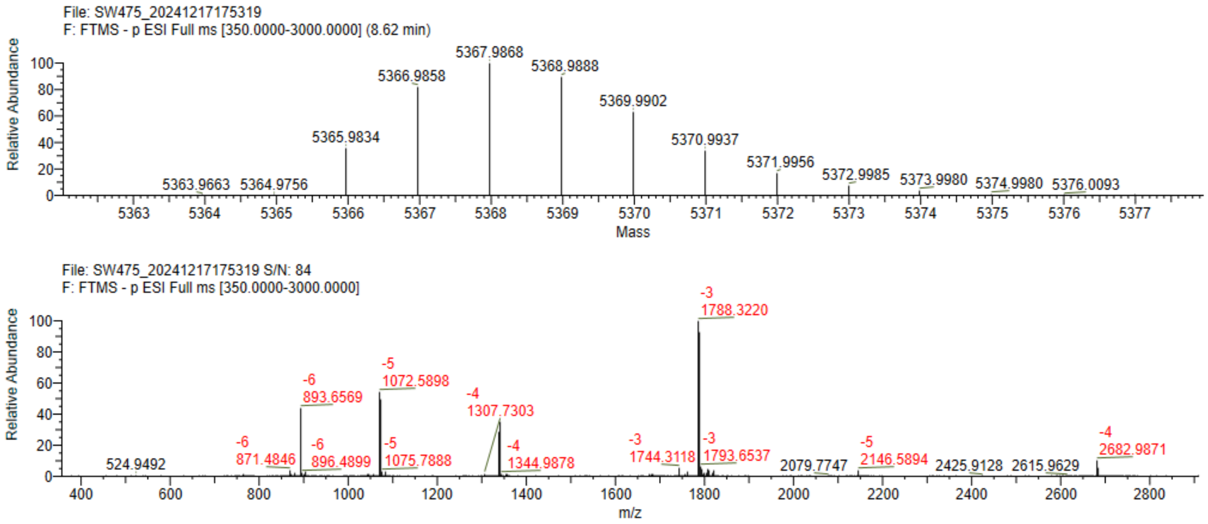

Figure S56: LC-MS spectra of conjugate **5k**.

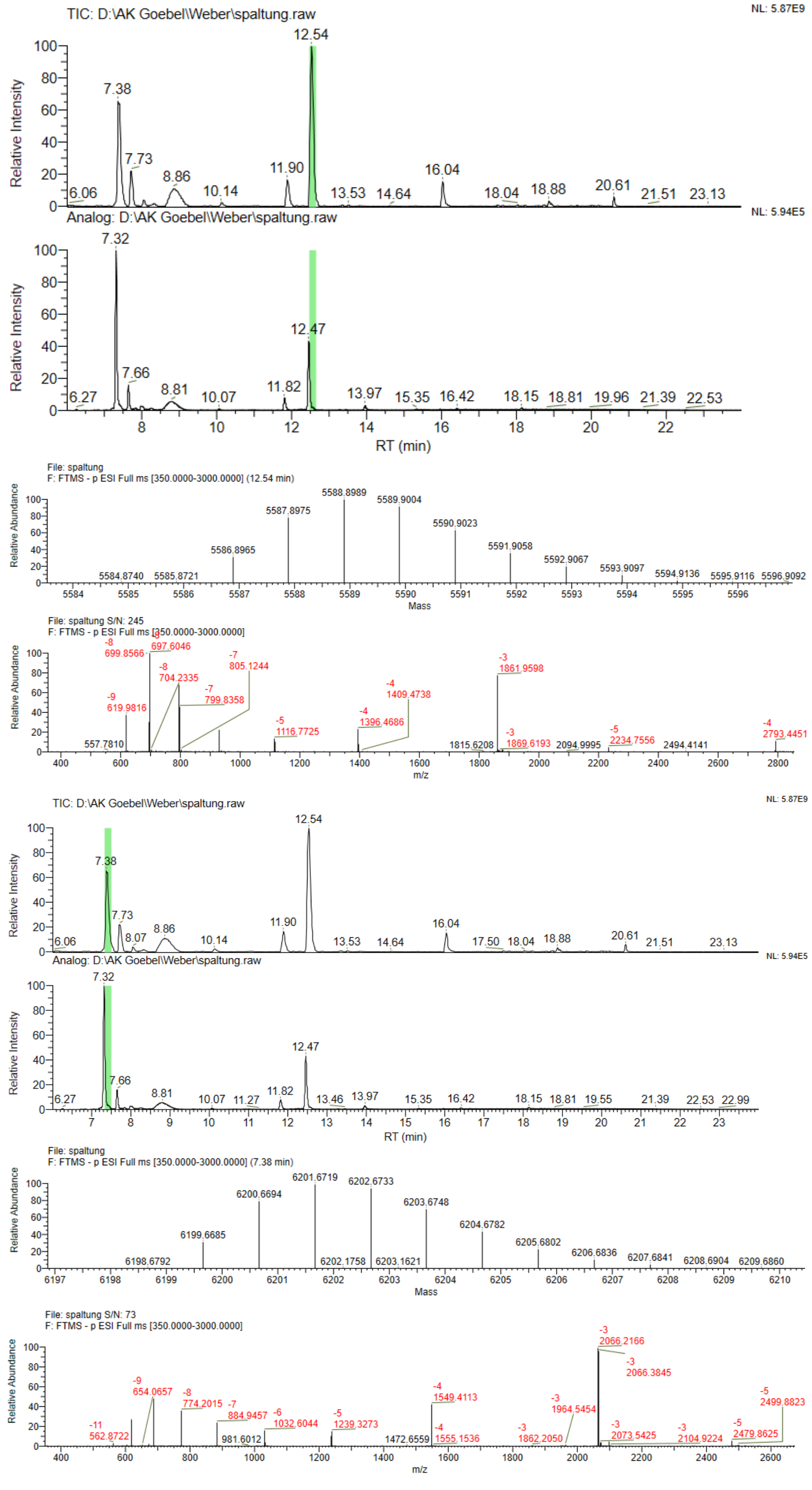

Figure S57. Cleavage of RNA **8** by conjugate **5k**, characterization of fragments by LC-MS. **Top**: Peak at 12.54 min has a deconvoluted mass of 5588.9 Da and corresponds to the 2,3-cyclic phosphate of Cy5-T_{10}-$^{5'}$CAACAU. **Bottom**: Peak at 7.38 min has a deconvoluted mass of 6201.7 Da corresponding to the fragment $^{5'}$UUACAACUCAUUCCAGTTTT. **Conditions**: 9 µM of RNA **8** and 3 µM **5k**, incubated for 20 h at 37 °C (50 mM Tris-buffer pH 8.0 without salt due to the incompatibility with MS).

Cleavage of 412mer transcript 9

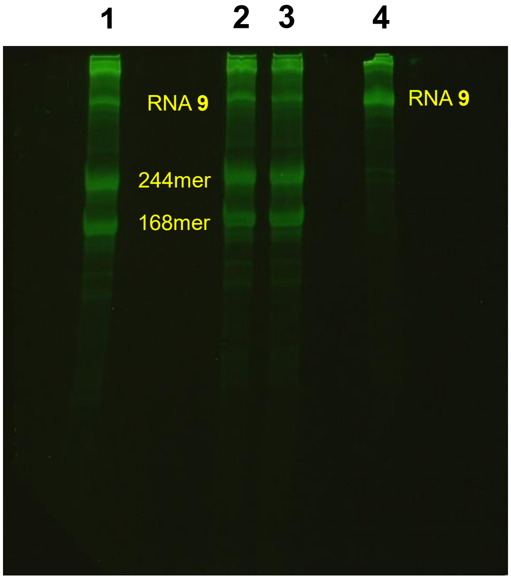

Figure S58: Cleavage of 412mer **9** with gapmer **10** and RNase H. 3 µM gapmer **10**, 2 µM RNA **9**, 0.5 U RNase H, 50 mM Tris-HCl, 75 mM KCl, 3 mM $MgCl_2$, 10 mM DTT, (pH 8.3), 37 °C, 3 minutes. **Lanes 1**, **2** and **3**: Three independent incubations, conditions as given above. **Lane 4**: 2 µM RNA **9**, 0.5 U RNase H, 50 mM Tris-HCl, 75 mM KCl, 3 mM $MgCl_2$, 10 mM DTT, (pH 8.3), 37 °C, 3 minutes. In absence of gapmer **10** the two faster running fragments are not formed.

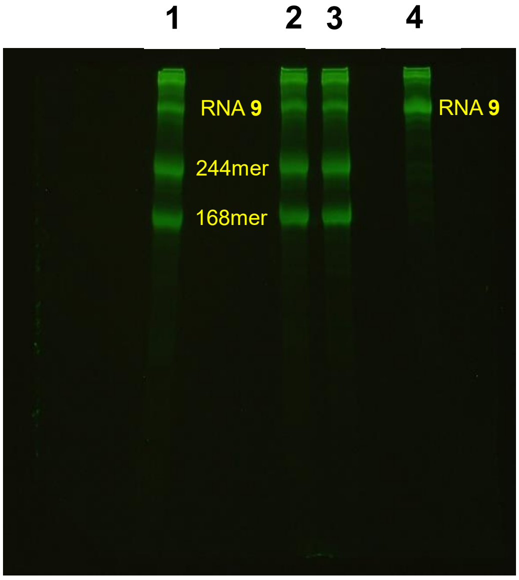

Figure S59: Cleavage of 412mer **9** with conjugate **5k**. 3 µM conjugate **5k**, 2 µM RNA **9**, 50 mM Tris-HCl, 100 mM NaCl, (pH 8.0), 37 °C, 4.5 h. **Lanes 1**, **2** and **3**: Three independent incubations, conditions as given above. **Lane 4**: 2 µM RNA **9**, 50 mM Tris-HCl, 100 mM NaCl (pH 8.0), 37 °C, 4.5 h. In absence of conjugate **5k** the two cleavage fragments are not formed.

Sequence of the 412mer RNA **9**:

$^{5'}$GGUCCACCUCCACAGCCUGUCGCCGGGGCCCAGCAAAUAGCAGCCUUUCUGGCAGGUCCUCCCCUCUC
UUGUCAGAUGCCCGAGGGAGGGGAAGCUUCUGUCUCCAGCUUCCCGAGUACCAGUGACACGUCUCGCCA
AGCAGGACAGUGCUUGAUACAGGAA<u>CAACAUUUACAACUCAUUCCAG</u>AUCCCAGGCCCCUGGAGGCUGCC
UCCCAACAGUGGGGAAGAGUGACUCUCCAGGGGUCCUAGGCCUCAACUCCUCCCAUAGAUACUCUCUUC
UUCUCAUAGGUGUCCAGCAUUGCUGGACGAUAUCGGAUCCCGGGCCCGUCGACUGCAGAGGCCUGCAUG
CAAGCUUGGCGUAAUCAUGGUCAUAGCUGUUUCCUGUGUGAAAUUGUUAUCCGCUCACAAUUCCACA$^{3'}$
